# Large‐Scale, Mechanically Robust, Solvent‐Resistant, and Antioxidant MXene‐Based Composites for Reliable Long‐Term Infrared Stealth

**DOI:** 10.1002/advs.202309392

**Published:** 2024-02-25

**Authors:** Bi‐Fan Guo, Ye‐Jun Wang, Cheng‐Fei Cao, Zhang‐Hao Qu, Jiang Song, Shi‐Neng Li, Jie‐Feng Gao, Pingan Song, Guo‐Dong Zhang, Yong‐Qian Shi, Long‐Cheng Tang

**Affiliations:** ^1^ College of Material, Chemistry and Chemical Engineering Key Laboratory of Organosilicon Chemistry and Material Technology of MoE Key Laboratory of Silicone Materials Technology of Zhejiang Province Hangzhou Normal University Hangzhou 311121 China; ^2^ Centre for Future Materials University of Southern Queensland Springfield 4300 Australia; ^3^ College of Chemistry and Materials Engineering Zhejiang A&F University Hangzhou 311300 China; ^4^ College of Chemistry and Chemical Engineering Yangzhou University Yangzhou Jiangsu 225002 China; ^5^ School of Agriculture and Environmental Science University of Southern Queensland Springfield 4300 Australia; ^6^ College of Environment and Safety Engineering Fuzhou University Fuzhou 350116 China

**Keywords:** infrared stealth, long‐term anti‐oxidation, micro‐/nanoarchitecture, MXene, weather resistance

## Abstract

MXene‐based thermal camouflage materials have gained increasing attention due to their low emissivity, however, the poor anti‐oxidation restricts their potential applications under complex environments. Various modification methods and strategies, e.g., the addition of antioxidant molecules and fillers have been developed to overcome this, but the realization of long‐term, reliable thermal camouflage using MXene network (coating) with excellent comprehensive performance remains a great challenge. Here, a MXene‐based hybrid network comodified with hyaluronic acid (HA) and hyperbranched polysiloxane (HSi) molecules is designed and fabricated. Notably, the presence of appreciated HA molecules restricts the oxidation of MXene sheets without altering infrared stealth performance, superior to other water‐soluble polymers; while the HSi molecules can act as efficient cross‐linking agents to generate strong interactions between MXene sheets and HA molecules. The optimized MXene/HA/HSi composites exhibit excellent mechanical flexibility (folded into crane structure), good water/solvent resistance, and long‐term stable thermal camouflage capability (with low infrared emissivity of ≈0.29). The long‐term thermal camouflage reliability (≈8 months) under various outdoor weathers and the scalable coating capability of the MXene‐coated textile enable them to disguise the IR signal of various targets in complex environments, indicating the great promise of achieved material for thermal camouflage, IR stealth, and counter surveillance.

## Graphical abstract

A high‐performance thermal camouflage material is designed and successfully fabricated by decorating MXene network with hyaluronic acid (HA) and hyperbranched polysiloxane (HSi). Besides excellent mid‐infrared (IR) thermal camouflage, such material also integrates multiple advantages into itself, including being large‐scale, mechanically flexible, weather‐resistant, and thus showing great potential for stealth applications.

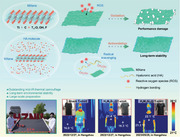

## 1 Introduction

With the evolution of nature over millions of years, many terrestrial or marine animals have evolved unique camouflage abilities that can almost perfectly integrate into the natural environment without being detected by natural enemies, such as chameleons, squids, octopuses, frogs, and etc.^[^ ^1^, ^2^, ^3^, ^4^ ^]^ It appeals to numerous researchers devoted to the development of camouflage technology from the inspiration of animals. Among them, if any temperature of the object is higher than the absolute zero degrees centigrade defined by thermodynamics, it will emit thermal radiation all the time, which will be easily captured and recognized by current infrared equipment. Therefore, infrared detection and anti‐detection have become the focus of military applications in various countries.^[^ ^5^, ^6^, ^7^, ^8^ ^]^ Typically, thermal camouflage stealth is realized by reducing the thermal radiation difference between the target and its surroundings.^[^ ^6^, ^9^ ^]^ There are two strategies to achieve thermal camouflage. One is to reduce the surface temperature (T) of the target, such as thermal insulation materials^[^ ^2^, ^6^, ^10^, ^11^, ^12^, ^13^ ^]^ and phase‐change materials (PCMs),^[^ ^14^ ^]^ and the other is to regulate the surface emissivity (e) of the target object, including the metal and its derivative film/coating.^[^ ^15^, ^16^, ^17^, ^18^, ^19^ ^]^ However, there are still many challenges in these infrared stealth materials, for example, difficulty in processing, easy corrosion, unstable thermal camouflage, mechanical brittle nature, and poor environmental reliability.^[^ ^16^, ^20^ ^]^

Ti_3_C_2_T*_x_* MXene (T denotes F, ─OH, and other terminal groups), a new 2D transition metal carbides and nitrides nanomaterial,^[^ ^21^, ^22^, ^23^ ^]^ has been widely researched, owing to its unique structure and outstanding properties, such as inorganic backbone, high mechanical strength, high electrical conductivity, rich surface chemistry, exceptional photothermal conversion property.^[^ ^24^, ^25^, ^26^, ^27^, ^28^ ^]^ During the past decade, MXene has been explored and applied in many emerging fields, such as sensing, optoelectronics, catalysis, biomedicine, energy storage, and etc.^[^ ^24^, ^29^, ^30^, ^31^, ^32^, ^33^, ^34^ ^]^ Among them, owing to its low mid‐IR emissivity (≈10%), the Ti_3_C_2_T*_x_* MXene paper demonstrated the excellent infrared (IR) stealth behavior of at different temperatures (from below −10 °C to over 500 °C),^[^ ^35^ ^]^ showing promising thermal camouflage application.^[^ ^36^ ^]^ Recently, Wang et al. made a lot of contributions in IR stealth of Ti_3_C_2_T*_x_* network from Mennen‐decorated porous polyethylene textile to dual‐mode porous polymeric/MXene films, further proving the excellent thermal camouflage performance of MXene composites.^[^ ^19^, ^37^, ^38^, ^39^ ^]^ Due to structural vacancies and atomic defects, MXene is easily eroded by O_2_ or H_2_O, and consequently, its superior performance will gradually disappear in complex environments.^[^ ^40^, ^41^, ^42^ ^]^ To solve this issue, some inspiring attempts have been implemented.^[^ ^43^, ^44^, ^45^, ^46^, ^47^ ^]^ For example, antioxidant MXene inks were developed by using the grafted polydopamine with a large number of catechol structures with scavenge free radical capability.^[^ ^48^ ^]^ Unfortunately, the surface modification of antioxidant molecules with high infrared emissivity inevitably induces deterioration of infrared stealth performance of MXene. Furthermore, the modified MXene‐based composite materials still hardly keep their long‐term structure stability in complex wet or water environments outdoor. Therefore, design and development of weather‐resistant MXene‐based thermal camouflage materials that meet the basic requirements of long‐term outdoor practical applications are strongly needed.

Hyaluronic acid (HA), a linear polysaccharide chain containing N‐acetylglucosamine and glucuronic acid, displays sensitive to reactive oxygen species (ROS), such as hydrogen peroxide, superoxide radical species, and the hydroxy radical species, and can scavenge ROS by the degradation of high molecular weight (HMW) HA.^[^ ^49^, ^50^, ^51^, ^52^ ^]^ During the degradation process, HMW HA may degrade into low molecular weight (LMW) hyaluronate fragments without destroying the structural units of HA.^[^ ^53^, ^54^ ^]^ Inspired with this, herein we report an MXene/polymer composite ink (referred to as MXene‐based ink) that possesses a dual‐network structure involving hydrogen bonding and chemical bonding through a reaction between HA and hyperbranched polysiloxane (HSi).^[^ ^55^, ^56^, ^57^ ^]^ Such dual‐network structure aims to enhance the oxidation resistance of the MXene‐based ink compared to pure MXene. MXene‐based papers, which are prepared by an environmentally friendly vacuum filtration method or low‐temperature evaporation, were then evaluated for their chemical structure, water resistance, and thermal camouflage properties. Remarkably, the thermal camouflage property and electrical conductivity of the MXene/HA paper exhibit great superiority over other MXene/polymer papers. Specifically, the optimized H_3_M_3_Si_2_ network shows a low mid‐IR emissivity of approximately 30% at 7–14 mm. Additionally, the H_3_M_3_Si_2_ ink was successfully employed to coat the surface of cotton textiles through a facile spraying technology, resulting in a black H_3_M_3_Si_2_@cotton textile. This coated textile displays exceptional environmental stability, enduring approximately eight months of exposure to nature, and has an excellent performance in terms of IR radiation stealth, which shows significant potential for weather‐resistant thermal camouflage applications.

## 2 Results and Discussion

### 2.1 Design and Preparation of Hybrid IR Stealth Material

**Figure 1:**
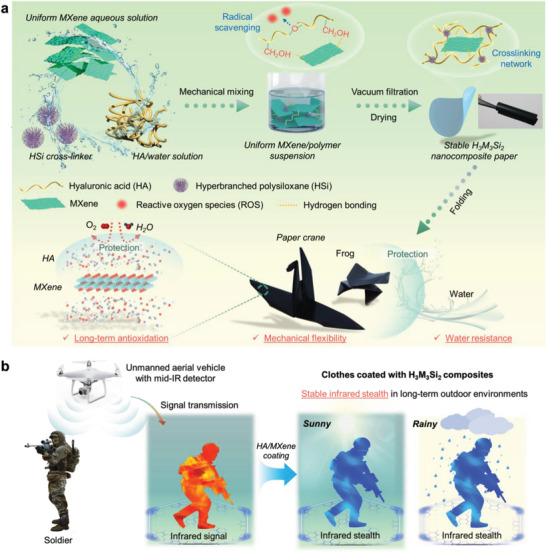
Design of polysiloxane modified HA/MXene network for infrared stealth in long‐term complex environments. a) Schematic illustration of preparation process of polysiloxane modified HA/MXene composite network, showing outstanding long‐term antioxidation, mechanical flexibility and water‐resistance properties. b) Schematic of stable infrared stealth scenarios of the optimized H_3_M_3_Si_2_ composite coating onto cotton textile, showing promising prospect for the consistency of target and background for soldier to avoid IR detection.

To create a thermal camouflage MXene‐based composite material that processes excellent environmental stability, three main ingredients are specifically required: i) low IR emissivity achieving to conceal objective from IR detection, ii) antioxidation ability to address oxidative instability of MXene, and iii) water‐resistance performance meeting long‐term application under outdoor weather situations (e.g., sunny, windy or rainy days). To meet these requirements, we selected high molecular weight HA as a scavenger of reactive oxygen species, HSi as a cross‐linking agent, and Ti_3_C_2_T*_x_* to fabricate a foldable H_3_M_3_Si_2_ paper (**Figure** **1**). Typically, few‐layered Ti_3_C_2_T*_x_* MXene sheets were obtained after etching and exfoliation of the MAX precursor (Figure S1a in Supporting Information), as confirmed by the SEM images and Tyndall phenomenon (Figure S1b–e, Supporting Information). Meanwhile, we synthesized the HSi cross‐linking agent using the synthesis route described in our previous work,^[^ ^58^ ^]^ which was verified by FTIR spectra and ^1^H spectra (Figure S2, Supporting Information). Under alkaline conditions, MXene sheets, HA molecules, and HSi were mixed to form a fluid H_3_M_3_Si_2_ ink through multiple physical and chemical interactions, and then the H_3_M_3_Si_2_ paper was prepared using a simple vacuum filtration method and low‐temperature aging to achieve excellent anti‐oxidation and water resistance (Figure 1a). Thermal camouflage textiles can be easily fabricated with the H_3_M_3_Si_2_ ink and they can evade the detection by a mid‐IR detector, showing a potential application in complex environmental conditions (Figure 1b). The detailed structure analysis and physical performance of H_3_M_3_Si_2_ network will be illustrated in the following sections.

### 2.2 Oxidation Behavior and Mechanism Analysis

**Figure 2:**
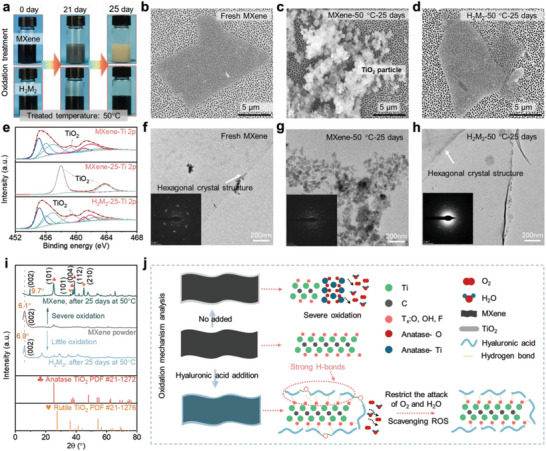
Long‐term antioxidation phenomenon and mechanism analysis of HA/MXene mixture. a) Comparison of optical images of pure MXene and HA/MXene (H_2_M_2_) aqueous solution after 50 °C aging treatment for 25 d, showing quite different oxidation behaviors. b–d) Typical SEM images, e) XPS Ti 2p spectra, f–h) TEM images and corresponding selected area electron diffraction (SAED) patterns of fresh MXene, MXene and H_2_M_2_ after aging for 25 d at 50 °C, confirming effective antioxidation after addition of HA molecules in MXene/water solution. i) XRD patterns of j) proposed antioxidation mechanism in the H_2_M_2_ composites.

To determine whether HA molecules can exhibit antioxidant performance, the MXene and H_2_M_2_ aqueous solution (MXene concentration of 3 mg mL^−1^) were subjected to a temperature of 50 °C for 25 d in an oven. Balancing the need for good oxidation resistance of MXene sheets with maintaining the excellent thermal camouflage of MXene‐based materials, an HA concentration of 3 mg mL^−1^ was selected. After 25 d, the MXene solution turned from dark black to yellow‐white, while the H_2_M_2_ solution remained dark green (**Figure** **2**a). Typical SEM images of fresh MXene, aged MXene, and aged H_2_M_2_ are shown in Figure 2b–d and Figure S3a–d (Supporting Information) revealing a clean surface of the fresh sheets (Figure 2b and Figure S3a, Supporting Information). After storing MXene in water at 50 °C for 21 d, it displayed a gray color (Figure 2a), and the SME image showed some fragmented sheets and nanoparticles (Figure S3c, Supporting Information). Continuing to age the MXene aqueous solution for an additional 4 d resulted in the complete transformation of the sheets into nanoparticles, as shown in Figure 2c,g and Figure S3d,e (Supporting Information). In contrast, the sheet structure and single crystal structure of the H_2_M_2_ were successfully retained after 25 d (Figure 2d,h and Figure S3b in Supporting Information), indicating that HA can effectively mitigate the MXene degradation in water and protect the MXene sheets from oxidation.

To further verify the anti‐oxidation property of HA, the chemical compositions of MXene and H_2_M_2_ aqueous solution after the aging process were characterized by X‐ray photoelectron (XPS) spectra, as depicted in Figure 2e. The Ti 2p spectrum of fresh MXene showed peaks of Ti 2p_3/2_ and Ti 2p_1/2_ observing within 452–468 eV. It was found that the MXene dispersion aged for 25 d displays two new peaks (458 and 464 eV) corresponding to TiO_2_, while the Ti 2p_3/2_ and Ti 2p_1/2_ peaks disappeared.^[^ ^43^, ^59^, ^60^, ^61^ ^]^ Although the intensity of TiO_2_ of H_2_M_2_ aged for 25 d was slightly stronger than in fresh MXene, H_2_M_2_ retained the peaks of Ti 2p_3/2_ and Ti 2p_1/2_ (Figure 2e). Furthermore, a hexagonal atomic arrangement observed in a selected area electron diffraction (SAED) pattern (inset of Figure 2f) confirms that the fresh MXene sheets is single crystalline, consistent with previous reports.^[^ ^62^ ^]^ Additionally, Raman spectra and X‐ray diffraction (XRD) patterns provided further evidence, as shown in Figure 2i and Figure S4 in Supporting Information. The aged H_2_M_2_ exhibited the characteristic MXene peaks (155, 398, and 630) in Raman spectra, while the aged MXene showed peaks (152, 262, 421, and 612) corresponding to TiO_2_. The XRD test shows consistent results, with the aged H_2_M_2_ remaining the characteristic MXene (002) peak, although the MXene sheets appeared slightly oxidization after 25 d at 50 °C (Figure 2i). However, the aged MXene displayed severe oxidation due to erosion from H_2_O and O_2_ resulting in the formation of TiO_2_ nanoparticles comprising anatase crystal (101, 004, 112) and rutile crystal (101, 210) (Figure 2i,e).

Both H_2_O and O_2_ molecules are responsible for the oxidizing Ti_3_C_2_T*_x_*. We reasonably speculate that the anti‐oxidation mechanism of MXene in the presence of HA can be divided into two reasons: i) the scavenging reactive free radical capability of HA, as mentioned in previous introductions;^[^ ^53^, ^54^ ^]^ ii) the shielding effect of the Ti_3_C_2_T*_x_* structure by HA molecule that associates with the sheet surface (Figure 2j). In the EDS test of MXene in an aqueous solution of HM (Figure S5 in Supporting Information), the enrichment phenomenon of N element on Ti_3_C_2_T*_x_* sheet indicates stronger adsorption between HA and MXene, attributable of hydrogen bonding and intermolecular forces.^[^ ^45^ ^]^ The formation of HA coating on the MXene surface is conductive for restricting the attack of H_2_O and O_2_ (Figure 2j). In addition, the EDS results show a sharp increase in oxygen (O) atomic content from 35.7% to 60.73% in aging MXene control, while the titanium (Ti), carbon (C), and fluorine (F) atomic content decreased (Figure S6, Supporting Information). In the H_2_M_2_ sample, the O atomic content in the Ti_3_C_2_T*_x_* sheet remains as expected. Surprisingly, the Ti and F atomic content increased from 31.07% and 9.71% to 32.61% and 15.17%, respectively, while the C atomic content decreases from 22.93% to 14.89%. This suggests that some HA molecule may detach from the Ti_3_C_2_T*_x_* sheet, which is likely due to the preferential scavenging of ROS in water by HA molecules (Figure S7, Supporting Information), leading to the formation of LWM HA fragments through degradation reactions.

### 2.3 Interaction Characterization and Mechanical Property

**Figure 3:**
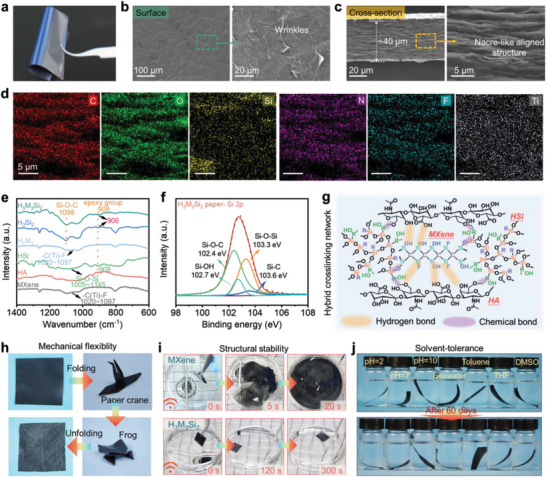
Structure characterization and interaction analysis in HA/MXene composites and their excellent mechanical flexibility and long‐term water resistance. a) Optical image, b,c) SEM images of the surface and the cross‐section, and d) EDS images of H_3_M_3_Si_2_ paper. e) FTIR spectrum of pure MXene, HA, HSi, H_2_M_2_, H_3_Si_2_ and H_3_M_3_Si_2_ composites, and f) XPS Si 2p spectra and g) interaction mechanism analysis in H_3_M_3_Si_2_ composites. h) Digital photograph of H_3_M_3_Si_2_ paper after multi‐step folding process, showing excellent mechanical flexibility and robustness. i) Comparison of structural stability of pure MXene and H_3_M_3_Si_2_ composite paper after ultrasonic treatment. j) Long‐term structure evolution of H_3_M_3_Si_2_ papers before and after being immersed in various solutions for 60 d, confirming outstanding water resistance.

Based on the aforementioned information, the water‐soluble nature of HA and MXene limits the outdoor applications of the H_2_M_2_ paper because of its water‐soluble nature. To address this issue, we introduce a hyperbranched polysiloxane (HSi) crosslinking agent with an epoxy group to construct a covalent bond during the drying process, achieving further stability of MXene‐based material. As shown in **Figure** **3**a–c, the cross‐section SEM images of H_3_M_3_Si_2_ paper via the vacuum filtration method indicate a nacre‐like aligned structure with a high level of orientation and rough wrinkled morphology and the C, O, Si, N, F, and Ti elements are well‐distributed in the H_3_M_3_Si_2_ paper (Figure 3d), demonstrating the uniform dispersion of the HA and HSi molecules in the final samples.

Fourier‐transform infrared spectroscopy (FTIR), X‐ray photoelectron spectroscopy (XPS), and ultrasonic treatment were employed to analyze the chemical structure and verify the existence of multiple interactions, e.g., hydrogen bonding and chemical bonding in the HA/MXene/HSi hybrid network. FTIR spectra in Figure 3e and Figure S8a (Supporting Information) reveal significant hydrophilic C═O groups at 1620 cm^−1^, 1680–1630 cm^−1^, and 1636 cm^−1^ for various materials (i.e., MXene, HA, and HSi, respectively). After drying, the peaks representing the stretch vibration of O─H slightly shift form ≈1633 to 1629 cm^−1^ in the H_2_M_2_ and H_3_M_3_Si_2_ papers, indicating the presence of strong hydrogen bonding between MXene sheets and HA molecules. This is also supported by a lower wavenumber (1620 cm^−1^) for the C═O peak of MXene when compared with the H_2_M_2_ and H_3_M_3_Si_2_ papers and the unchanged C═O peak of the H_3_Si_2_ paper also implies the absence of hydrogen bonds between HSi and HA molecules (Figure S8a, Supporting Information). Moreover, a new peak appear at 1098 cm^−1^ emerges, which is assigned to the Si─O─C bond, and the bending vibration peak of C─O─C (derived from epoxy) shifts from 908 cm^−1^ to 906 cm^−1^, which is likely due to the hydroxyl condensation reaction between Si─OH group of HSi and C─OH group of HA molecules, as well as the ring‐opening reaction between the epoxy of HSi and the CH_2_─OH group of HSi (Figure S10, Supporting Information).^[^ ^55^, ^56^, ^57^, ^63^ ^]^ Furthermore, XPS results in Figure S8 (Supporting Information) indicate that compared to O1s MXene, the H_2_M_2_ paper presents a higher bonding energy feature of C─Ti─OH (530.9 eV) and C─O (533.2 eV) due to the formation of strong hydrogen bonding.^[^ ^64^ ^]^ Similar covalent bonding between HA molecules or MXene sheets and HSi is also obtained in the H_3_M_3_Si_2_ paper, as indicated by the appearance of Si─O─Si (532.5 eV and 103.3 eV), Si─O─Ti (531.0 eV), and Si─O─C (102.4 eV) in the XPS O1s and Si2p spectra (Figure 3f and Figure S9a, Supporting Information).

Besides, compared with the sharp diffraction peak at ≈6.9° corresponding to the interlayer spacing of ≈1.28 nm for pure MXene paper, the H_2_M_2_ paper displays an increased interlayer spacing (5.9°, ≈1.50 nm) due to the introduction of the HA molecule intercalated into the adjacent MXene sheets (Figure S11, Supporting Information).^[^ ^45^, ^65^ ^]^ This is attributed to the formation of hydrogen bonding between the HA molecule and the MXene sheet. In addition, the interlayer spacing value of the H_3_M_3_Si_2_ paper is consistent with that of H_2_M_2_ paper (i.e., 5.9°, ≈1.50 nm), implying that HSi would react with the MXene sheet and HA molecules, as presented in Figure 3g. Owing to the interplay of multiple interactions, the H_3_M_3_Si_2_ paper can be folded into the complex shapes like paper cranes and frogs (Figure 3h). The typical strain–stress curve of various papers is shown Figure S12 (Supporting Information). The tensile strength of MXene paper is lower (≈5 MPa) compared to other MXene‐based paper samples due to the presence of voids formed from during the vacuum filtration method used for its preparation (Figure S13, Supporting Information).^[^ ^66^ ^]^ However, incorporating HA and HSi into the MXene network are effective to enhance the tensile strength of the MXene/HA/HSi paper, for instance, ≈54 MPa for the optimized H_3_M_3_Si_2_ paper. Along with the increase in the tensile strength, the elongation at break and toughness are also improved (Figure S12d, Supporting Information). On the other hand, excessive addition of HSi molecules causes a decrease in the tensile strength of MXene‐based paper, which would be attributed to the brittleness caused by excessive cross‐linking points (Figure S12c, Supporting Information).

The ultrasonic treatment of the samples in water can provide further evidence for the formation of multiple interactions in the optimized H_3_M_3_Si_2_ paper. As shown in Figure 3i and Figure S14a,b (Supporting Information), both MXene paper and H_2_M_2_ paper readily disperse and colorize the entire aqueous phase during ultrasonic process, suggest the poor interfacial interactions among MXene, HA and HSi. In contrast, the H_3_Si_2_ paper exhibits a swelling phenomenon due to the presence of the covalent bonding (Movie S1, Supporting Information). Remarkably, the H_3_M_3_Si_2_ paper maintains its original size and structure for a duration of 30 min (Movie S2, Supporting Information), and UV–Vis spectroscopy of H_3_M_3_Si_2_ paper test group shows no absorbance peaks at ≈790 nm for MXene,^[^ ^43^ ^]^ further confirming the existence of multiple physical and chemical bonds (Figure S14c, Supporting Information). In addition, although the MXene paper exhibits a water contact angle of ≈106° higher than most MXene‐based composite paper (Figure S15a, Supporting Information), it is unable to withstand immersion in water; while the H_3_M_3_Si_2_ papers can keep their structure integrity even after being immersed for 60 d in different acid/alkaline solutions and organic solvents with various solubility parameters (Figure 3j and Figure S15b, Supporting Information). Overall, owing to the formation of strong physical and chemical interactions in the MXene/HA/HSi network, the optimized H_3_M_3_Si_2_ paper demonstrates the outstanding structure stability, excellent mechanical flexibility and favorable water tolerance in harsh solutions.

### 2.4 Infrared Stealth Property and Analysis of MXene‐Based Interconnected Network

**Figure 4:**
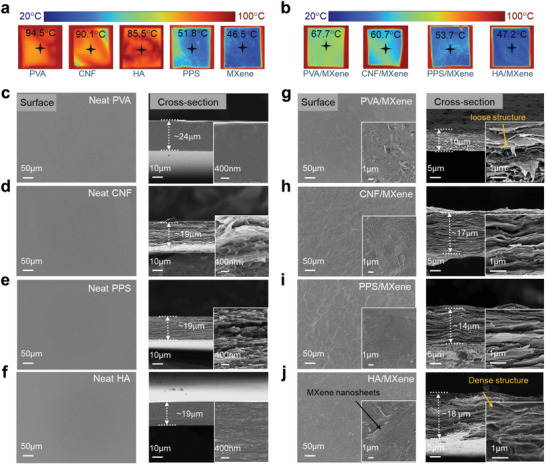
Comparison of various MXene/polymer composite for thermal camouflage performance. a,b) IR thermal image of PVA, CNF, PPS, HA, and pure MXene, and their PVA‐M, CNF‐M, PSS‐M and HA‐M composite papers covered on an object with onto a hot surface with a fixed temperature of 100 °C, showing stable thermal camouflage performance in HA/MXene composites in comparison with other MXene/polymer composites. c–j) SEM images of surface and cross‐section of different polymer papers and their MXene/polymer composites.

Normally, the infrared stealth performance of materials is related to the emissivity of their surface,^[^ ^35^, ^36^ ^]^ and they commonly exist in the form of composite materials in real‐world applications. However, the infrared emissivity of coatings/films increases with high concentrations of polymer material (also known as film former) with a high IR emissivity.^[^ ^7^ ^]^ Thus, it is still a critical challenge to obtain MXene‐based polymer composites with stable infrared stealth property, especially under complex outdoor environments. **Figure** **4**a,b shows the infrared thermographic images of different polymer papers and their MXene/polymer composite papers that were subjected onto a hot stage at 100 °C for 30 min. As expected, the PVA (polyvinyl alcohol), cellulose nanofiber (CNF) or HA polymer papers at a fixed thickness value of ≈20 µm exhibit poor infrared stealth properties since the surface temperature values are close to that of the hot stage (Figure 4a). In comparison, the PST (poly(3, 4‐ethylene dioxythiophene): poly(styrene sulfonate) and MXene papers with high conductivities display obvious low surface temperature under the sample test conditions, i.e., 51.8 °C and 46.5 °C, respectively. After combined the polymer with the MXene at a fixed weight ratio, although the surface temperature values of the PVA/MXene and CNF/MXene composites are much lower than those of pure polymer materials, they are much higher than the pure PST and MXene papers, as shown in Figure 4b. Interestingly, an unprecedented phenomenon was observed in the HA/MXene composites, and the radiation surface temperature of 47.2 °C is very close to that of pure MXene paper, superior to other polymer/MXene composite papers. This demonstrates that the exceptional infrared stealth property obtained in the HA/MXene composites.

The well‐known Hagen‐Rubens equation^[^ ^67^ ^]^ can be used to evaluate the infrared emissivity (*e*) of the metal film, and the stealth capability of materials in the mid‐IR range is positively correlated with their electrical properties.^[^ ^68^ ^]^ MXene, being a derivative of the metal, could potentially follow this principle as well. As shown in Figure S15 (Supporting Information), the electrical conductivity and the surface electrical resistance of MXene‐based papers exhibit a similar trend to the aforementioned thermal camouflage results. The electrical properties of HA/MXene composites (electrical conductivity: ≈250 S cm^−1^ and surface electrical resistance: ≈50 Ω, Figure S16a, Supporting Information) are superior to those of other composite materials, which is the highest electrical conductivity among all the polymer/MXene composites. XRD results confirm that compared with the interlayer spacing of ≈1.28 nm (6.9°) in pure MXene paper, the HA/MXene, PST/MXene, and CNF/MXene papers exhibit increased interlayer spacing (e.g., ≈1.50 nm (5.9°), 1.36 nm (6.5°), and 1.34 nm (6.6°), respectively) due to hydrogen bonding between MXene and polymer molecules (Figure S16b, Supporting Information),^[^ ^65^ ^]^ but the HA molecules has little influence on the MXene network, thus producing the effectively conductive path in the HA/MXene composites (Figure S17, Supporting Information). SEM images of the cross‐section of all neat polymer papers (Figure 4c–f), the cross‐section morphologies of the polymer/MXene composites are similar as pure MXene paper (Figure 4g–j and Figure S18, Supporting Information), showing typical multilayered structure features. Careful observation suggests that the PVA, CNF and PTS molecules intercalated into the MXene sheets reduce the thickness of pure MXene paper at the same MXene weight, e.g. from ≈19 µm to be ≈10 µm for the PVA/MXene in Figure 4g. However, the presence of HA molecules seems to form dense structure, which does not alter the MXene network, which is also supported the wrinkled structure observed in the sample surface for both pure MXene and the HA/MXene shown in Figure S18a (Supporting Information) and Figure 4j.

**Figure 5:**
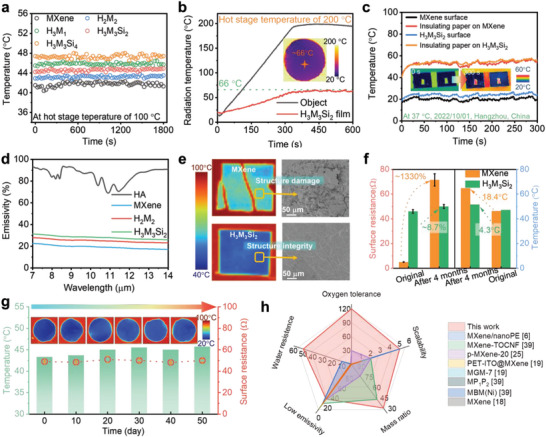
Thermal camouflage capability of HA/MXene composites. a) Comparison of thermal camouflage behaviors of various MXene‐based papers under 100 °C. b) Thermal camouflage behavior of H_3_M_3_Si_2_ papers under 200 °C and corresponding IR images. c) Thermal camouflage test of H_3_M_3_Si_2_ paper in the piratical environment (autumn in Hangzhou). d) Mid‐IR emissivity of HA, MXene, H_2_M_2_ and H_3_M_3_Si_2_ papers. e,f) Long‐term oxidation measure of MXene‐based papers: IR thermal images, SEM images, and IR radiation temperature and surface resistance. g) Long‐term stability of H_3_M_3_Si_2_ paper after being immersed in seawater for 50 d. h) Comparison of various properties of H_3_M_3_Si_2_ with other reported MXene‐based IR stealth materials.

Notably, various HA/MXene papers show excellent thermal camouflage capability, and their surface radiation temperature remains lower than 46 °C for 30 min on the 100 °C heating stage (**Figure** **5**a, S19, Supporting Information). Surprisingly, the radiation temperature of H_3_M_3_Si_2_ paper is ≈45 °C close to pristine MXene paper suggesting an outstanding thermal camouflage ability despite the effect of HSi on this property (Figure 5a). Although the thermal stability property of H_3_M_3_Si_2_ paper is worse than that of MXene paper, it can meet most application environments under 200 °C (Figure S20, Supporting Information). The thermal radiation temperature of the H_3_M_3_Si_2_ paper covered on various temperatures hot stage (e.g., 100 °C, 150 °C, and 200 °C, respectively) always remains at a low value with increasing the heating stage temperature (Figure 5b and Figure S21, Supporting Information), demonstrating the superior thermal camouflage performance and thermal stability of the H_3_M_3_Si_2_ paper. In addition, the H_3_M_3_Si_2_ paper attached onto human palm could reduce the skin radiation temperature (33.7 °C) to 27.2 °C, and the H_3_M_3_Si_2_ paper can be cut to the letters “HZNU” showing a potential of the H_3_M_3_Si_2_ paper for transmitting IR signals (Figures S22,S23, Supporting Information).

Due to the good conductive path in our MXene‐based papers, they exhibit low electrical resistance and high EMI SE (>30 dB), surpassing the higher industrial standard of 20 dB (Figure S24a,b, Supporting Information).^[^ ^69^ ^]^ Moreover, the radiation temperature of the H_3_M_3_Si_2_ paper measured ≈43 °C when the applied voltage was 3 V (Figure S24c, Supporting Information). This temperature does not represent the actual temperature because of its low‐emissivity in mid‐IR. To determine the actual temperature during the Joule heating process, an insulating paper was affixed (≈0.98 emissivity) on the H_3_M_3_Si_2_ paper. The measured temperature was ≈120 °C, approximately 80 °C higher than the disguised radiation temperature. Thus, the H_3_M_3_Si_2_ paper is suitable for electronic components used in weapon equipment and fulfills the requirements for heating the body and maintaining infrared stealth in cold environments.

Shown in Figure 5c (with Figure S25, Supporting Information serving as an illustration picture) demonstrates that the thermal radiation temperature of the H_3_M_3_Si_2_ paper is approximately 23 °C, which is lower than its actual temperature (≈56 °C) in autumn, aligning with the findings for MXene. Under the summer control group (Figure S26, Supporting Information), the H_3_M_3_Si_2_ paper still displays the same phenomenon, suggesting the disguised photothermal capability of the H_3_M_3_Si_2_ paper. To further evaluate the IR stealth property of the composite materials, we measured their mid‐IR reflectivity and transmissivity (Figure S27, Supporting Information), and calculated their emissivity at 7–14 µm (Figure 5d) according to the thermal radiation relation *ε* = 1‐*r*‐τ (where *ε*, *r*, and τ represent emissivity, reflectivity, and transmissivity, respectively). Both the H_2_M_2_ and H_3_M_3_Si_2_ paper show respectively low IR emissivity of ≈26% and ≈29% at 7–14 µm, close to the pure MXene paper (20%), even though the IR emissivity of the HA paper shows a high IR emissivity of ≈80% (Figure 5d). This is mainly because the compact structure in the H_3_M_3_Si_2_ paper ensures outstanding electrical performance, favoring IR stealth capability.

The antioxidation property of HA in practical application were evaluated using H_3_M_3_Si_2_ paper prepared through a simple low‐temperature evaporation process. As shown in Figure S28 (Supporting Information), the surface of MXene paper turned gray‐white due to the attack of the O_2_ molecule after storing outdoors for 4 months. This damage compromised the IR stealth performance of the MXene paper, resulting in an increase in surface resistance and surface thermal radiation temperature by 1335% (from 5 to 71.5 Ω) and 18.4 °C, respectively (Figure S29, Supporting Information and Figure 5e,f). It is attributed to the oxidation and fragmentation behavior of MXene nanosheets under the erosion of O_2_ molecules for aged MXene paper (Figure 5e and Figures S30,S31, Supporting Information). In contrast, the H_3_M_3_Si_2_ paper remains almost unaffected and exhibited outstanding environmental stability properties and maintains a smooth microscopic surface structure throughout the aging process (Figure 5e and Figures S28,S29, Supporting Information), and the element ratio on the surface remained unchanged (Figure S31, Supporting Information). These observations further support the notion that the introduction of HA molecule is beneficial for enhancing the stability of MXene in natural environments. It is worth noting that the stability of MXene in paper formation is superior to that in water aqueous. This can be attributed to two factors: 1) there are more ROS present in water compared to air, and 2) the intermolecular forces in paper formation are stronger than that those in aqueous solution formation. Moreover, the saltwater resistance is also a critical concern for MXene‐based materials in outdoor applications. Remarkably, the thermal camouflage performance and electrical properties of the H_3_M_3_Si_2_ paper remained stable even after continuous exposure to 3.5 wt% saltwater for 50 continuous days (Figure 5g). Furthermore, the surface morphology, each element ratio, and crystalline structure of the H_3_M_3_Si_2_ paper was unchanged after storing it in salt water for 50 d, (Figures S32,S33, Supporting Information), indicating its excellent outdoor environmental stability.

Additionally, Figure 5h and Table S1 (Supporting Information) provide a comparison of the typical properties of our H_3_M_3_Si_2_ material and MXene‐based thermal camouflage materials reported in previous studies.^[^ ^19^, ^36^, ^37^, ^38^, ^48^, ^70^, ^71^, ^72^, ^73^, ^74^ ^]^ It is worth noting that the MXene‐based materials achieving low IR emissivity through the lay‐by‐lay method rely on the MXene layer located on the outer surface layer of the composite. In such materials, the MXene content is defined as 100%. However, the combination of outstanding oxygen tolerance, scalability, low IR emissivity, low MXene content, and excellent water resistance properties has not been observed in any of the previously reported MXene‐based IR stealth materials. These exceptional features, coupled with the facile fabrication process, make our H_3_M_3_Si_2_ materials highly promising for practical applications in thermal camouflage materials that require outdoor environmental adaptability and reliability.

### 2.5 Demonstration of Expanded Applications

**Figure 6:**
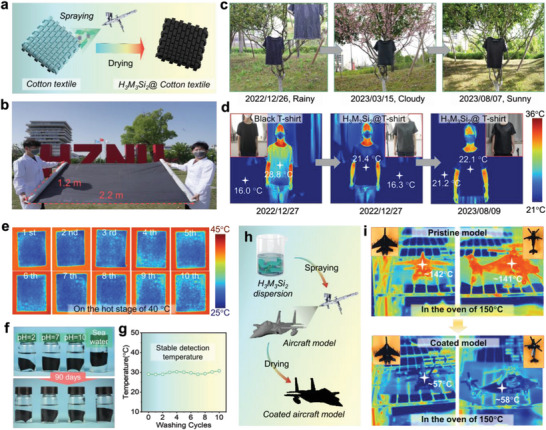
Demonstration of scalable production and thermal camouflage application scenarios. a) Schematic of fabrication process of H_3_M_3_Si_2_ coated T‐shirt (named H_3_M_3_Si_2_ @T). b) Photograph of the H_3_M_3_Si_2_@cotton textile at large‐scale production. c) Digital image of H_3_M_3_Si_2_@cotton cloth after exposing outdoor for about 8 months. d) Optical and (I) IR photographs of a person who wore black T‐shirt (left, named black‐T), H_3_M_3_Si_2_ @T‐shirt (middle), and H_3_M_3_Si_2_ @T‐shirt (right) stored in environment for ≈8 months. e) Thermal infrared images of H_3_M_3_Si_2_ coated textile before/after washing with different times, and f) Structure stability of H_3_M_3_Si_2_ fabric before and after immersed in various solution for 90 d. g) Corresponding surface radiation temperature of H_3_M_3_Si_2_ textile after washing different cycles. h) Schematic preparation of aircraft model coated with the H_3_M_3_Si_2_ composites, and i) corresponding the thermal camouflage test under high‐temperature environments.

Despite undergoing a chemical reaction during magnetic stirring process (Figure 1a), the H_3_M_3_Si_2_ ink maintains superior fluidity. Consequently, we proposed a straightforward a‐step process (**Figure** **6**a) for manufacturing the H_3_M_3_Si_2_@Cotton textile by spraying on commercially available cotton textiles. A roll of H_3_M_3_Si_2_@Cotton textile 1.2 m in wide and 2.2 m in length was prepared using the same scalable approach (Figure 6b). The morphology of the cotton fiber is shown in Figure S34a (Supporting Information) (left), displaying a characteristic rough surface with a diameter of approximately 12 mm. In contrast, the cotton fiber of the H_3_M_3_Si_2_@Cotton textile exhibits a thin cover (≈13 mm) with folds (Figure S34a, Supporting Information, right), and its surface exhibits the well‐distributed C, N, O, Si, F, and Ti elements (Figure S34b, Supporting Information) due to the affinity between cotton and H_3_M_3_Si_2_ materials and potential chemical reaction between the ─CH_2_OH groups of cotton fiber and HSi molecules. Thus, the H_3_M_3_Si_2_ coating exhibits excellent weather‐resistant stability after long‐term outdoor exposure. After exposure to varied weather conditions outdoors (e.g., sunny, rainy, and cloudy, as shown in Figure 6c) for approximately 8 months, these H_3_M_3_Si_2_ composite samples still maintained excellent IR stealth performance. The optical and IR images of an individual wearing a white T‐shirt (right) or a black H_3_M_3_Si_2_@T‐shirt (middle) in Figure 6d demonstrate that the temperature reading of the H_3_M_3_Si_2_@T in the IR image was only 21.4 °C, considerably lower than the body temperature of 36 °C, and the surface temperature of the black T‐shirt which was measured at 28.8 °C, appearing nearly as cold as the environment (16.3 °C). Further, the wash resistance of textile materials is a critical concern. To simulate machine washing, we performed a wash test in dynamic water with a magnet rotor stirring at 600 rpm speed for 10 min (Movie S3, Supporting Information). Regardless of the number of wash tests, the surface radiation temperature of the H_3_M_3_Si_2_@cotton textile remains around 30 °C (Figure 6e,g), suggesting its potential for commercial use. Furthermore, the structure of H_3_M_3_Si_2_@T remains stable and resistant to water during ultrasonic exposure for 10 min without detachment of H_3_M_3_Si_2_ from the cotton textile, implying excellent adhesion between H_3_M_3_Si_2_ and cotton textiles (Figure S35 and Movie S4, Supporting Information). Moreover, the H_3_M_3_Si_2_@T shows exceptional environmental stability, even in seawater, acidic, and alkaline aqueous solutions (Figure 6f). This outcome highlights the material's durability and resistance to adverse weather conditions, making it suitable for prolonged outdoor applications, confirming the H_3_M_3_Si_2_ with its low IR emissivity, which could be employed as an IR camouflage coating to conceal human bodies from IR thermal detection.

Shown in Figure 6h is the surface temperature of airplane models coated by H_3_M_3_Si_2_ materials. The surface temperature was approximately 84 °C and lower than that of the uncovered airplane models in the oven at 150 °C (Figure 6i), further exemplifying the potential application of the H_3_M_3_Si_2_ materials in the coating field. Moreover, by combining low emissive materials with thermal insulating aerogel materials, we can achieve a composite material with superior thermal camouflage properties. The fabrication process for the H_3_M_3_Si_2_/glass fiber@silicone aerogel composites involved wrapping and spraying steps, as shown in Figure S36 (Supporting Information). Consequently, the H_3_M_3_Si_2_/GF@A composites demonstrate exceptional IR stealth properties, culminating in a significant reduction of object radiation from ≈450 °C to ≈93 °C, which shows the potential thermal camouflage applications in some emerging fields.

## 3 Conclusions

We describe a simple and environmentally friendly processing approach to fabricate the MXene‐based composites with weather‐resistant, long‐term, and reliable thermal camouflage performance, which is achieved by incorporating HA and HSi molecules to comodify the MXene network. Typically, the addition of appreciate HA greatly restricts the easy oxidation of MXene, and the HSi molecules act as efficient cross‐linking agents to generate well cross‐linked hybrid network. As a result, the optimized H_3_M_3_Si_2_ composites demonstrate outstanding long‐term anti‐oxidation and exceptional structure stability in various complex solution environments, e.g., water, salt–water, acid/alkaline aqueous solutions, as well as organic solvents. Further, such MXene‐based composites also display low infrared emissivity, disguised Joule heating, and photothermal capability. Based on the H_3_M_3_Si_2_ aqueous solution sprayed onto cotton textiles using an extremely simple and scalable process, the H_3_M_3_Si_2_ coated cotton composites exhibit good mechanical flexibility, reliable IR stealth performance, and excellent durability after repeated washing and outdoor environmental exposure, enabling them can be used as ideal weather‐resistant thermal camouflage candidate for military and security applications.

## 4 Experimental Section

### Materials

Ti_3_AlC_2_ precursor (MAX) was purchased from Jilin Technology Co., Ltd. The poly(3, 4‐ ethylene dioxythiophene): poly(styrene sulfonate) was obtained from Shanghai Ouyi Organic Optoelectronic Materials Co., Ltd., China. Cellulose nanofibrils and hyperbranched polysiloxane were synthesized in the laboratory. HA with a molecular weight of 200 000, lithium fluoride salt (LiF) with a purity of 98%, hydrochloric acid (HCl) with a concentration of 35%, polyvinyl alcohol with a molecular weight of 6500, and other reagents were purchased from Sinopharm Chemical Reagent Co., Ltd. (China). All chemicals were used without purification.

### Preparation of MXene Aqueous Solution

Ti_3_C_2_T*_x_* MXene sheets were synthesized by the procedure described in previous works.^[^ ^24^, ^25^, ^75^, ^76^ ^]^ Initially, MAX was added to a solution consisting of 30 mL volume and 9 mL of HCl, containing 3 g of LiF. The mixture was stirred at 35 °C for 48 h. After the reaction, the suspension was subjected to sonication for 0.5 h to obtain the dispersed Ti_3_C_2_T*_x_* nanosheets. Subsequently, the dispersion was washed and centrifuged repeatedly until the pH reached approximately 6. Finally, a dark green MXene/water dispersion with a certain concentration was obtained, and the resulting sediment was dispersed in 100 mL of deionized water (DI).

### Fabrication of MXene, Different Polymer, and Various MXene/Polymer Papers

First, a specific amount of water‐soluble CNF, PVA, PTS, or HA molecules were individually added to the MXene/water solution (with a concentration of 5 mg mL^−1^). These mixtures were uniformly obtained after magnetic stirring for 5 h. Subsequently, the suspension was transferred to a mold container and placed in an oven at 50 °C for approximately 12 h for low‐temperature drying. Following this drying process, various MXene/polymer papers were obtained, which were named HA/MXene, PVA/MXene, CNF/MXene, and PPS/MXene, respectively.

### Preparation of HM, HSi and HMSi Papers

Typically, a MXene/water solution with a concentration of 5 mg mL^−1^ or a specific amount of hyperbranched polysiloxane, containing vinyl and epoxy groups, was added to the HA/water solution with a concentration of 10 mg mL^−1^, all while stirring for 5 h. This process yielded the formation of HM, HSi, and HMSi inks. These inks were then used to create HM, HSi, and HMSi papers through vacuum filtration of the aforementioned uniform suspension. The resulting papers were subsequently dried in an oven at 50 °C for approximately 12 h. For ease of reference, the composite materials were labeled as H*_x_* M*_y_*, H*_x_* Si*_y_*, or H*_x_* M*_y_* Si*_z_*, wherein *x*/*y*/*z* represents the mass ratio of the respective components. For instance, H_3_M_3_Si_2_ denotes the nanocomposite paper consisting of 3 parts per hundred (phr) of HA, 3 phr of MXene, and 2 phr of HSi.

### Preparation of H_3_M_3_Si_2_@Cotton

The H_3_M_3_Si_2_ mixture was prepared following the previously described procedure. Subsequently, the mixture was applied to the surface of cotton using the spray‐coating method. The resulting coated cotton was identified as H_3_M_3_Si_2_@Cotton. It was then subjected to an oven at 50 °C for approximately 1 h to facilitate drying. Prior to the spray‐coating step, the H_3_M_3_Si_2_ solution was stirred for 10 min.

### Characterizations Methods

The detailed characterization methods are provided in Supporting Information.

## Conflict of Interest

The authors declare no conflict of interest.

## Author Contributions

B.‐F.G. and Y.‐J.W. contributed equally to this work. Investigation: B.F.G., Y.J.W., and C.F.C.; resources, data curation, writing—original draft, B.F.G., Y.J.W., and L.C.T.; conceptualization, methodology, supervision, L.C.T.; writing—review & editing, C.F.C. and L.C.T.; validation, software, B.F.G., Y.J.W., C.F.C., Z.H.Q., J.S., S.N.L., and G.D.Z.; resources, validation, P.S. and Y.Q.S.

## Supporting information

Supporting Information

Supplemental Movie 1

Supplemental Movie 2

Supplemental Movie 3

Supplemental Movie 4

## Data Availability

The data that support the findings of this study are available in the Supporting Information of this article.
