# Supplementary material for: Large‐Scale, Mechanically Robust, Solvent‐Resistant, and Antioxidant MXene‐Based Composites for Reliable Long‐Term Infrared Stealth
Source: Adv Sci (Weinh). 2024 Feb 25;11(17):2309392. doi: 10.1002/advs.202309392 (PMC11077694; doi:10.1002/advs.202309392)
Supplement: Supplementary file 1 — Supporting Information [file ADVS-11-2309392-s002.pdf]

ADVANCED SCIENCE
Open Access

Supporting Information

for *Adv. Sci.*, DOI 10.1002/advs.202309392

Large-Scale, Mechanically Robust, Solvent-Resistant, and Antioxidant MXene-Based Composites for Reliable Long-Term Infrared Stealth

Bi-Fan Guo, Ye-Jun Wang, Cheng-Fei Cao, Zhang-Hao Qu, Jiang Song, Shi-Neng Li, Jie-Feng Gao, Pingan Song, Guo-Dong Zhang, Yong-Qian Shi and Long-Cheng Tang**

Supplementary Materials for

Large-scale, mechanically robust, solvent-resistant, and antioxidant MXene-based composites for reliable long-term infrared stealth

Bi-Fan Guo [a, ǂ], Ye-Jun Wang [a, ǂ], Cheng-Fei Cao [a, b, *], Zhang-Hao Qu [a], Jiang Song [a], Shi-Neng Li [c], Jie-Feng Gao [d], Pingan Song [b, e], Guo-Dong Zhang [a], Yong-Qian Shi [f], Long-Cheng Tang [a, *]

[a] *College of Material, Chemistry and Chemical Engineering, Key Laboratory of Organosilicon Chemistry and Material Technology of MoE, Key Laboratory of Silicone Materials Technology of Zhejiang Province, Hangzhou Normal University, Hangzhou 311121, China*

[b] *Centre for Future Materials, University of Southern Queensland, Springfield 4300, Australia*

[c] *College of Chemistry and Materials Engineering, Zhejiang A&F University, Hangzhou 311300, China*

[d] *College of Chemistry and Chemical Engineering, Yangzhou University, Yangzhou, Jiangsu 225002, China*

[e] *School of Agriculture and Environmental Science, University of Southern Queensland, Springfield 4300, Australia*

[f] *College of Environment and Safety Engineering, Fuzhou University, Fuzhou 350116, China*

[ǂ] These authors authors contributed equally to this work.

* Correspondence should be addressed:

Email: cheng-fei.cao@usq.edu.au (C.F. C.); Email: lctang@hznu.edu.cn. (L.C. T.)

Table of Contents

Characterizations

Figures S1-S36

Tables S1 and Movies S1-S4

Experimental Section

Characterizations

The morphology and microstructure of MXene and various samples were analyzed using scanning electron microscopy (SEM) with an energy-dispersive spectrometer (EDS) on a Sigma-500, ZEISS instrument. Transmission electron microscopy (TEM) images were obtained using a Talos F200X G2 instrument with an accelerating voltage of 200 kV. The chemical compositions and structure of the materials were analyzed using a Nicolet 7000 Fourier-transform infrared (FT-IR) spectrometer and a VG Scientific ESCALab 220I-XL X-ray photoelectron spectrometer (XPS), respectively. The water contact angle of the samples was measured at room temperature using a DSA30 CA analyzer from Kruss, Germany. X-ray diffraction (XRD) analysis was performed using a Rigaku D/Max 2550 V X-ray diffractor with a 2θ range of 5° to 80°. The oxidation behavior of MXene and MXene composites was analyzed via Raman spectroscopy using a spectrometer from Bruker Instruments, Germany, and a universal electricity meter. The electrical conductivity of the samples was measured using a standard four-probe tester (ST2722-SD). The infrared (IR) reflectivity (r) and transmittance (t) were measured using an FTIR spectrometer (Nicolet iS50) equipped with an infrared integrating sphere, while the IR emissivity (e) was calculated using the equation $e=1-r-t$. The electrical conductivity and electromagnetic interference (EMI) shielding performance were measured in the frequency range of 8.2~12.4 GHz using an Agilent vector network analyzer (PNA-N5244A). The 1H cross-polarization magic angle spinning (CP/MAS) nuclear magnetic resonance (NMR) measurements were conducted on a 400 MHz NMR spectrometer from Bruker Corp., Germany. The uniaxial stress-strain tests were performed using a dynamic mechanical thermal analyzer (DMA-Q800) from TA Instruments, America. The thermal camouflage property of MXene-based papers was evaluated using infrared thermal imagers (FLIR E60 and FOTRIC 320Pro, America). The thermal performance of the samples was characterized using thermogravimetric analysis (TA Instruments Q500, America) under an air atmosphere with a heating rate of 10 °C/min from room temperature to 750 °C.

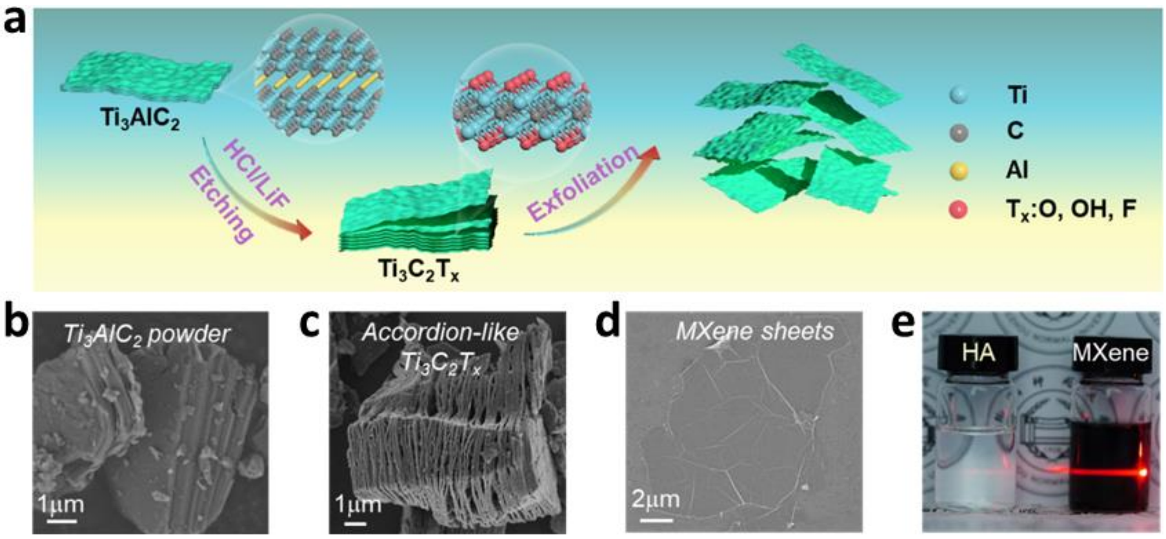

Figure S1. (a) Photographs showing the fabrication process of MXene *via* the etching and delamination method. SEM images of (b) Ti_3AlC_2 powder, (c) accordion-like $Ti_3C_2T_x$ and (d) MXene sheets. (e) Digital image of transparent HA (left) and MXene solvent dispersion (right).

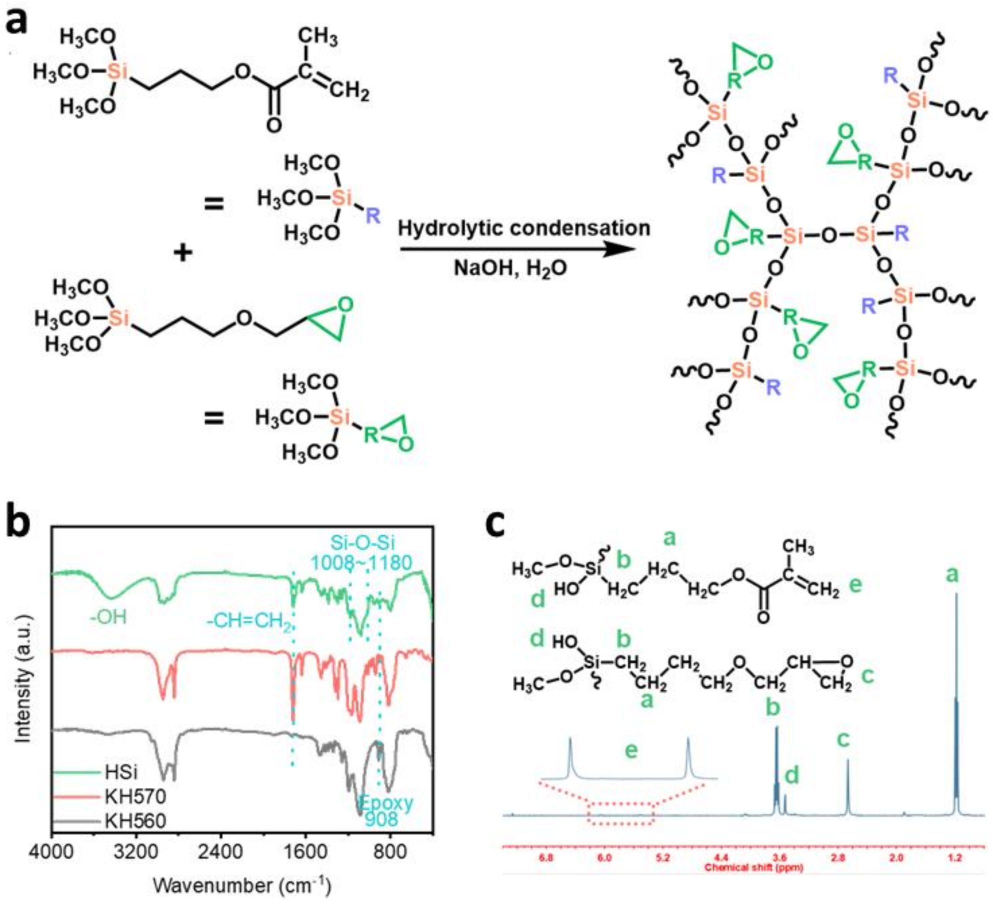

Figure S2. (a) Synthesis of hyperbranched polysiloxane (HSi). (b) FTIR spectra of KH560, KH570, and HSi. (c) ^{1}H spectra of HSi.

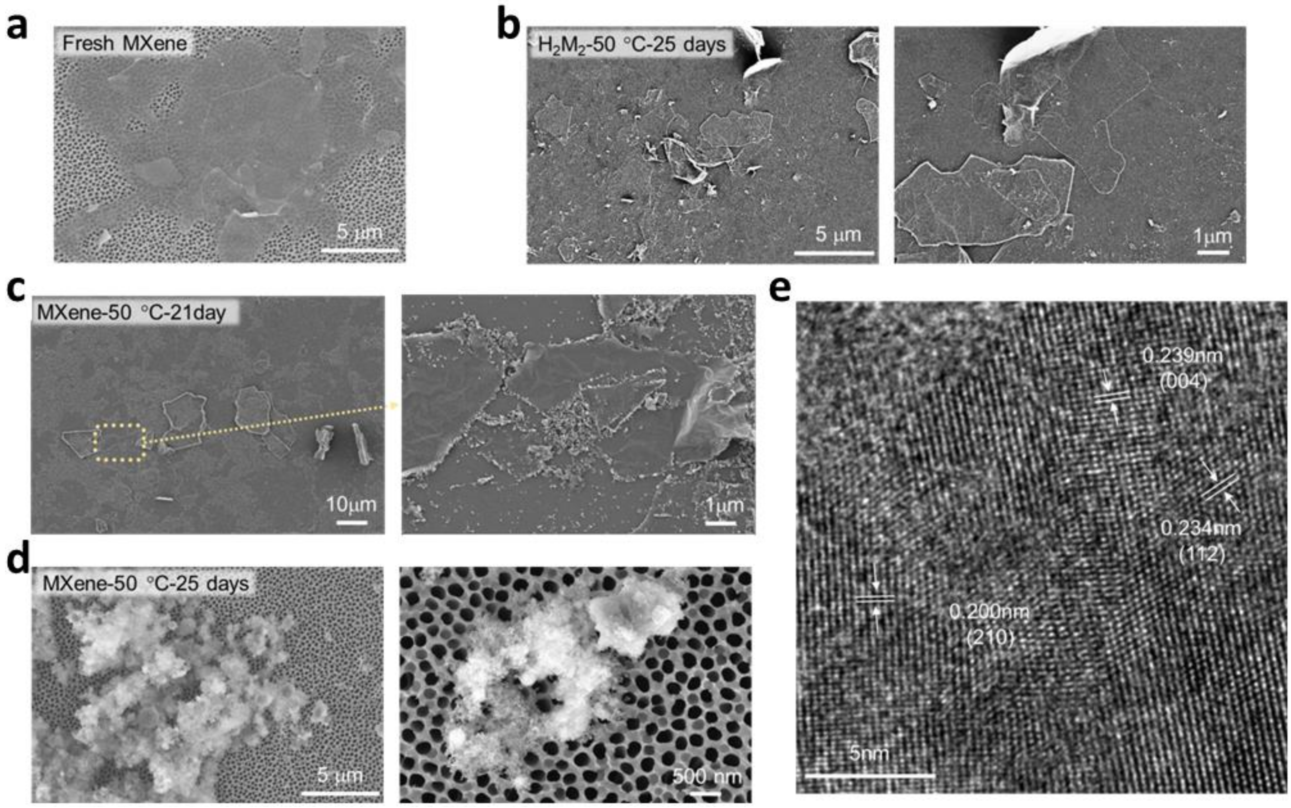

Figure S3. SEM image of (a) fresh MXene nanosheets, (b) H_2M_2 nanosheets aged 25 days, (c) MXene aged 21 days and (d) MXene aged 25 days. (e) TEM image of MXene aged 25 days.

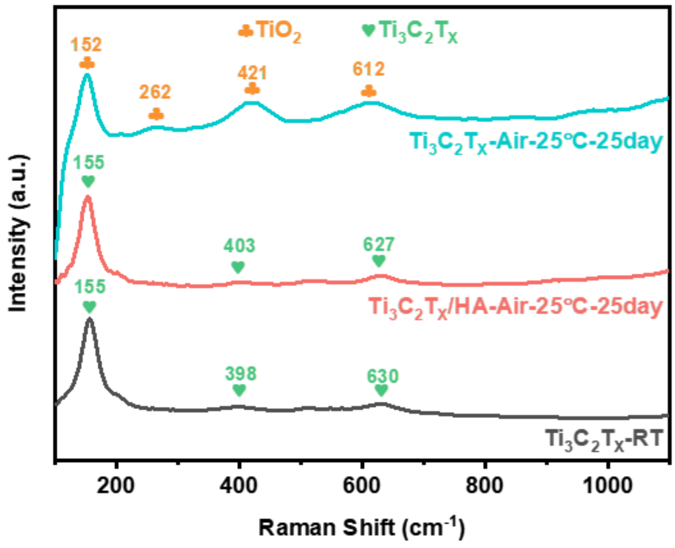

Figure S4. Raman spectra for fresh MXene, MXene after aging treatment, and MXene/HA mixture after aging treatment.

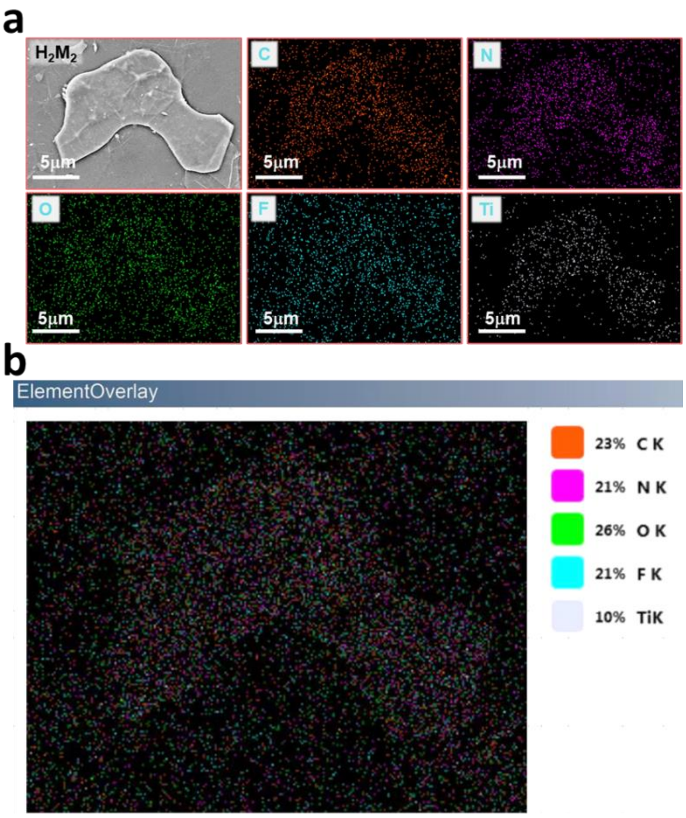

Figure S5. (a) Mapping test of a MXene/HA mixture dispersed on a silicon wafer and (b) the corresponding to overall element image and element proportion.

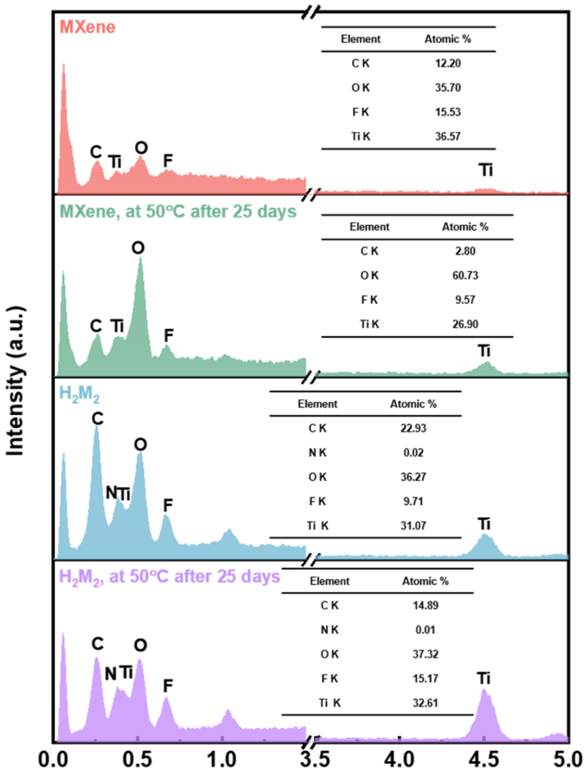

Figure S6. EDS results of MXene and MXene/HA mixture dispersed on silicon wafer before and after

aging treatment.

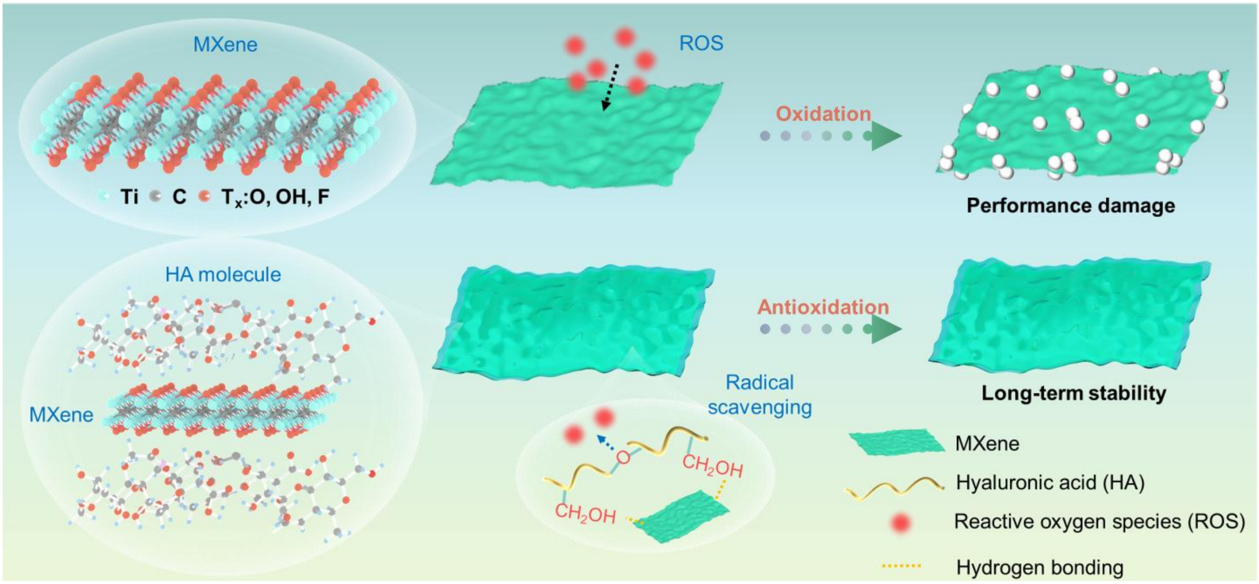

Figure S7. Oxidation process of MXene and MXene/HA composites.

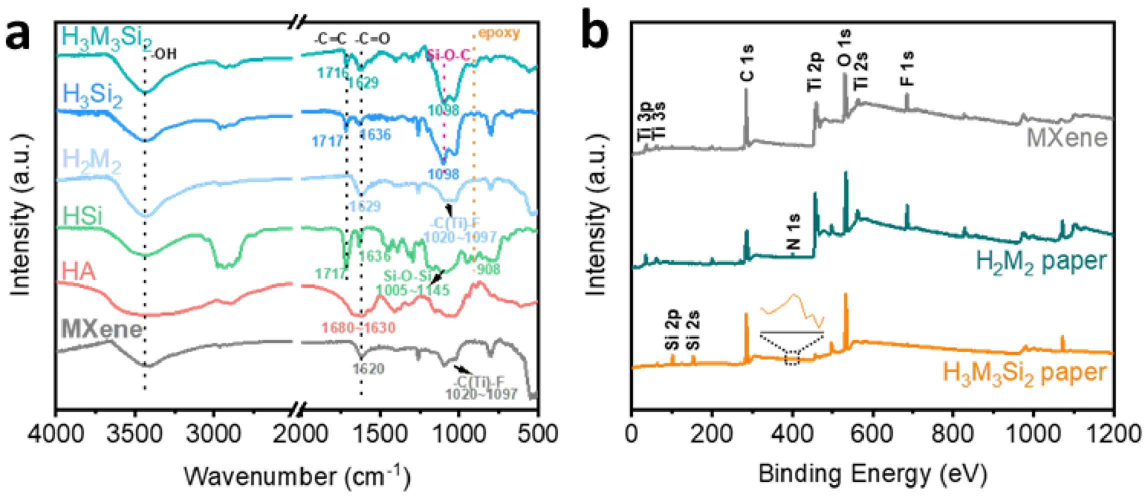

Figure S8 (a) FTIR (4000-500 cm^{-1}) of MXene, HA, HSi, H_2M_2, H_3Si_2 and $H_3M_3Si_2$. (b) XPS results of MXene paper, H_2M_2 paper and $H_3M_3Si_2$ paper.

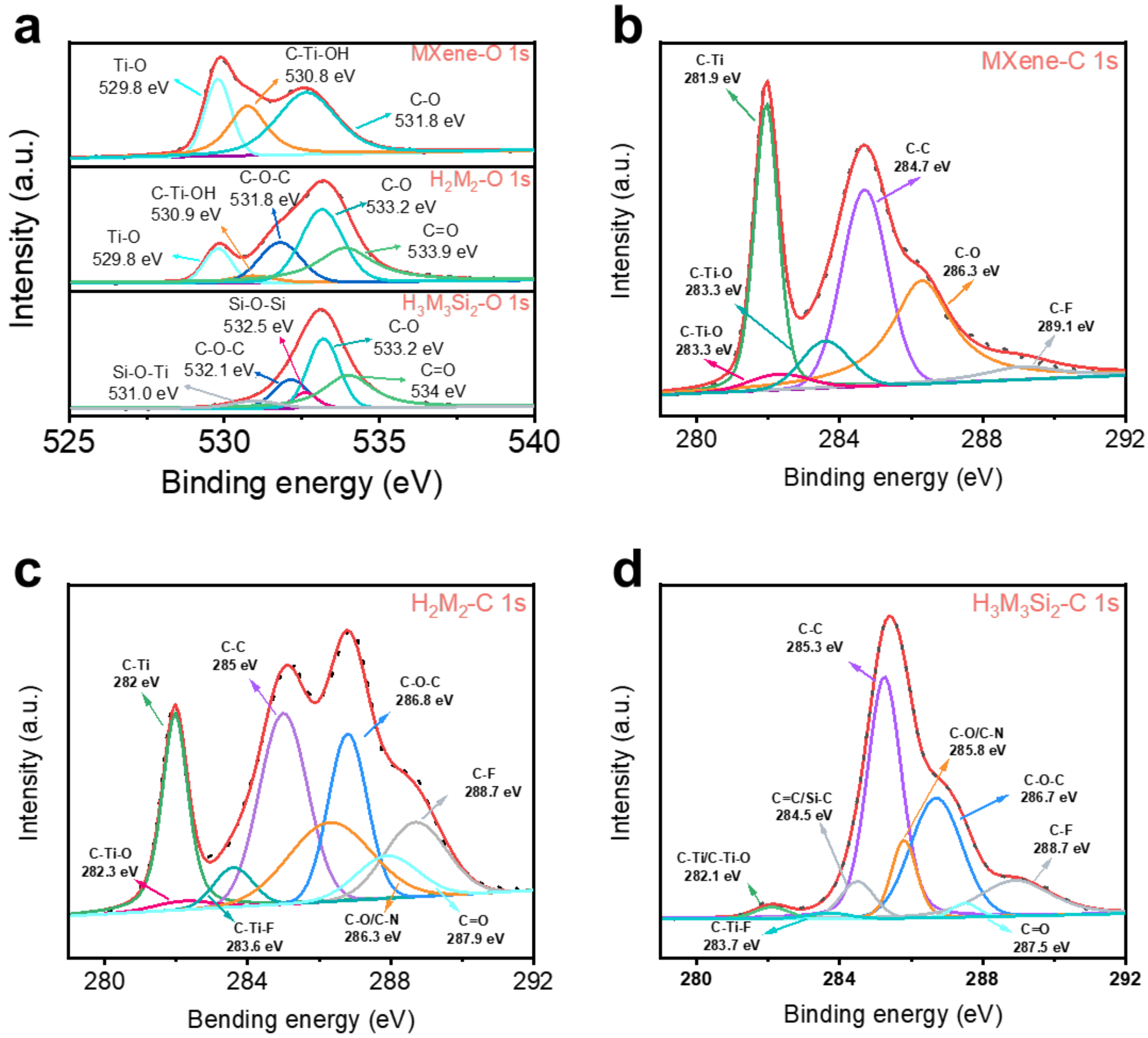

Figure S9. (a) XPS O 1s spectra of MXene, H_2M_2 and $H_3M_3Si_2$ papers. XPS C 1s spectra of (b) MXene paper, (c) H_2M_2 paper and (d) $H_3M_3Si_2$ paper.

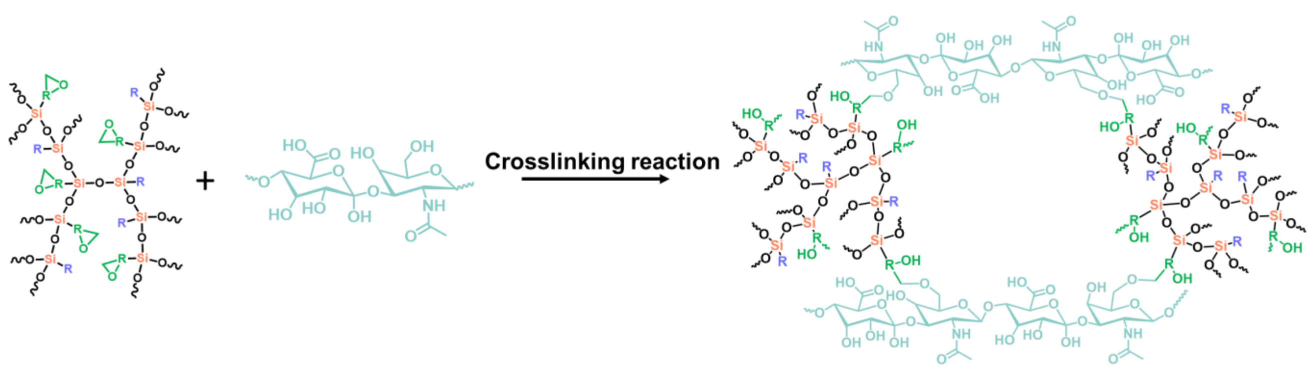

Figure S10. Chemical reaction between HA and HSi.

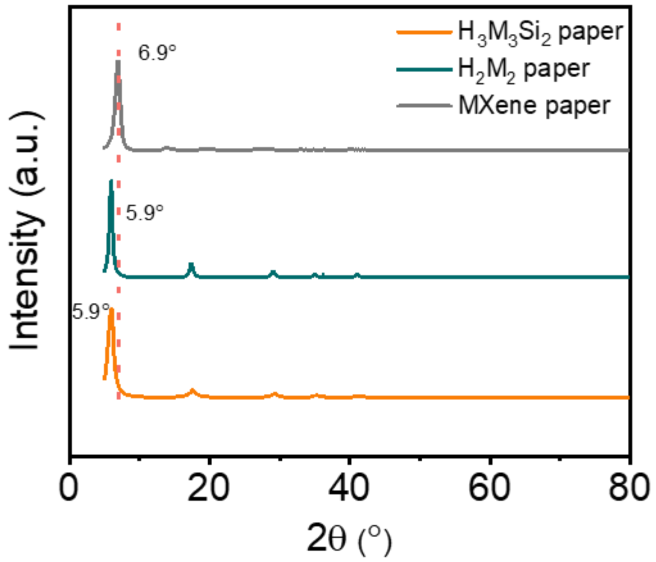

Figure S11. XRD patterns various papers samples.

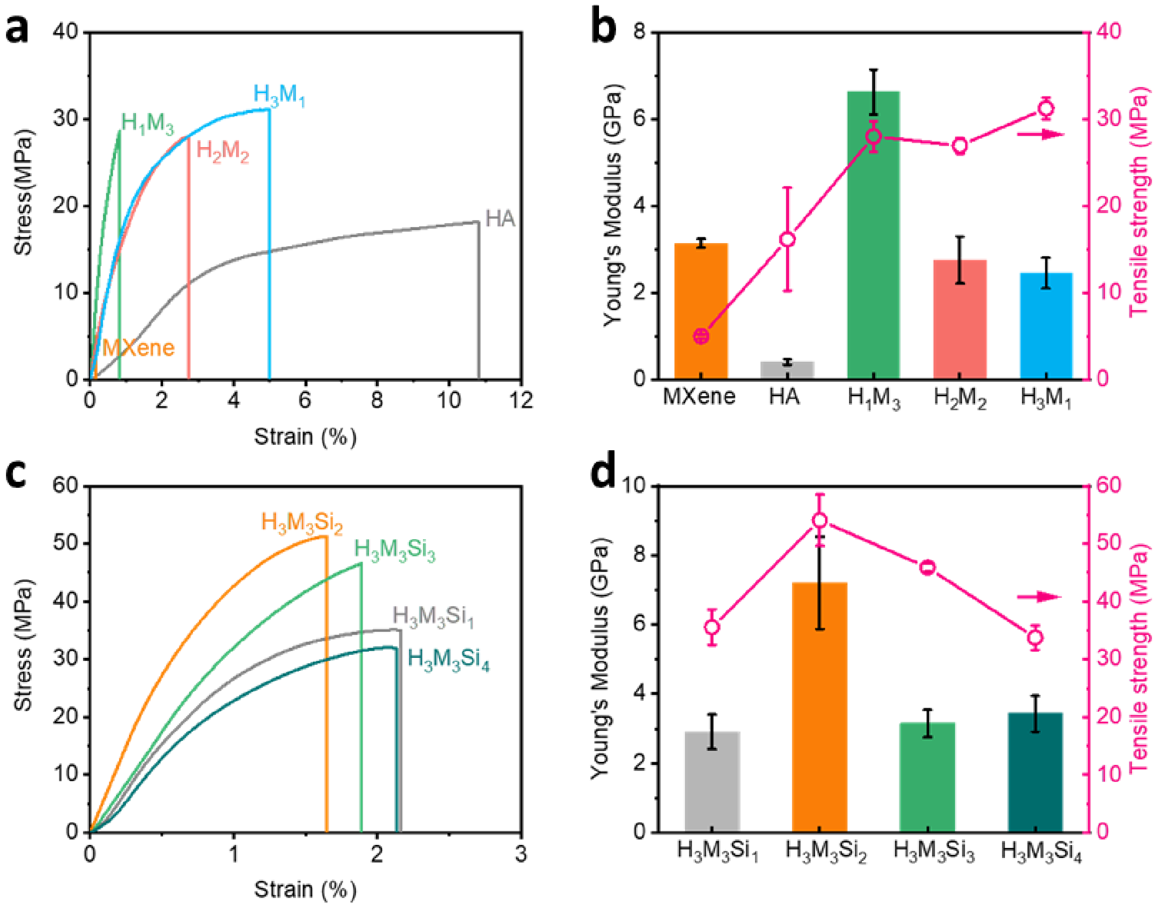

Figure S12. (a, c) Stress-strain, (b, d) Young's modulus and tensile strength.

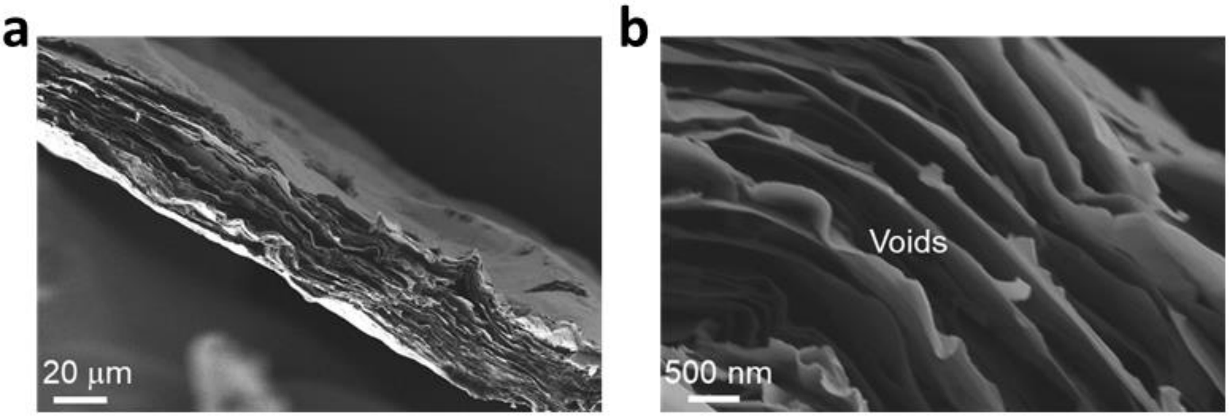

Figure S13. (a, b) SEM images of crossing-section of MXene paper with different magnifications.

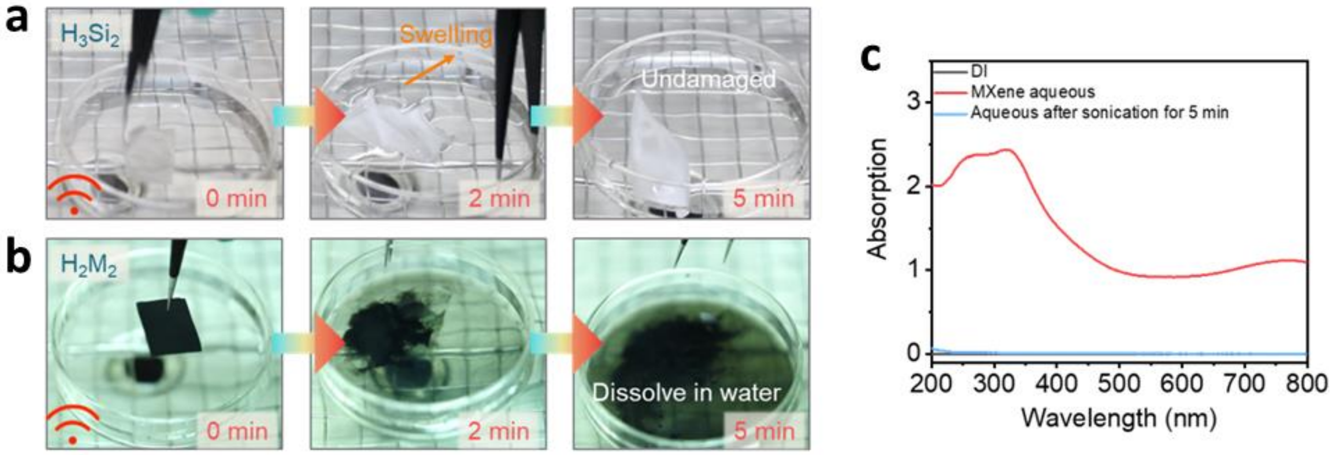

Figure S14. (a) Treating pristine H_3Si_2 paper and (b) H_2M_2 paper under ultrasonic circumstances. (c) UV-vis spectra for DI, pristine MXene dispersion and solution treated with ultrasonic in $H_3M_3Si_2$ group.

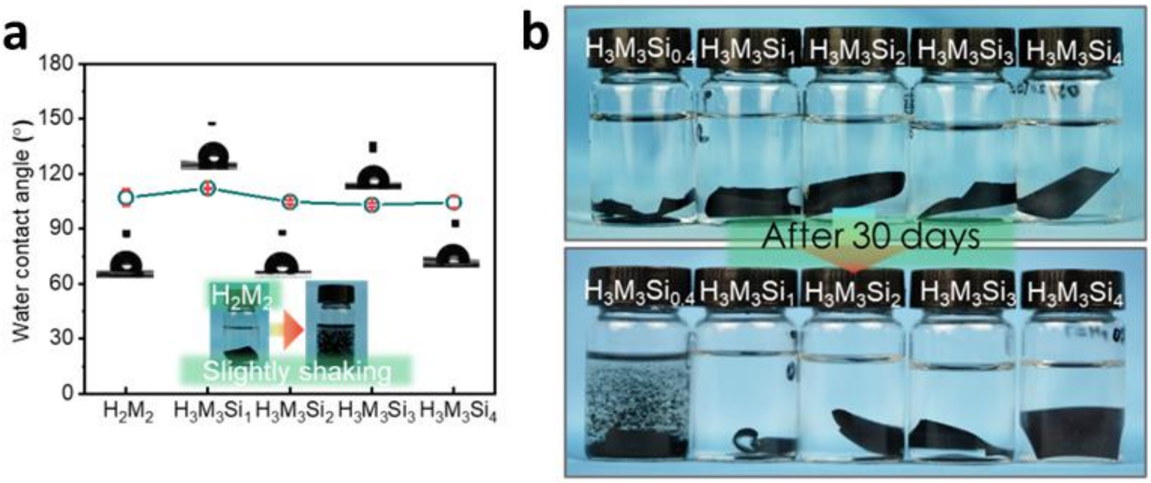

Figure S15. (a) Water contact angle for HM and HMSi nanocomposite papers. (b) Structure stability of various HMSi papers before and after being immersed in water for 30 days.

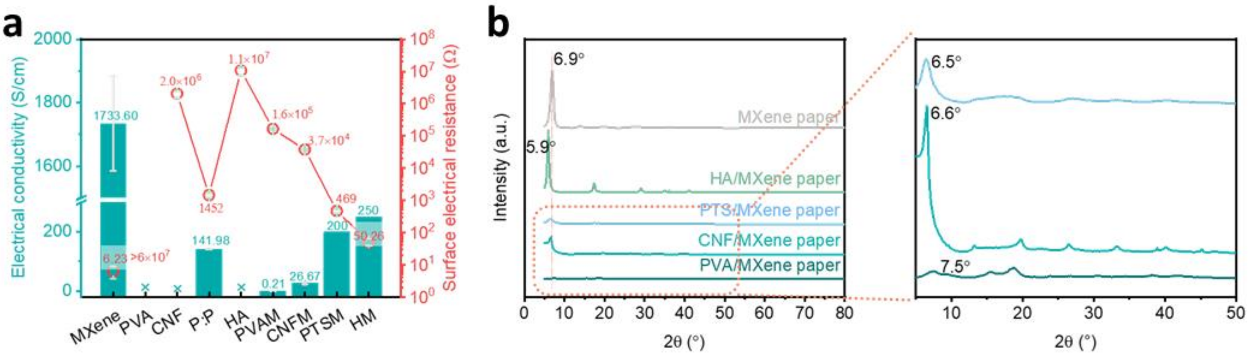

Figure S16. (a) Electrical conductivity, surface electrical resistance, and (b) XRD patterns of MXene, different polymer and various MXene/polymer papers.

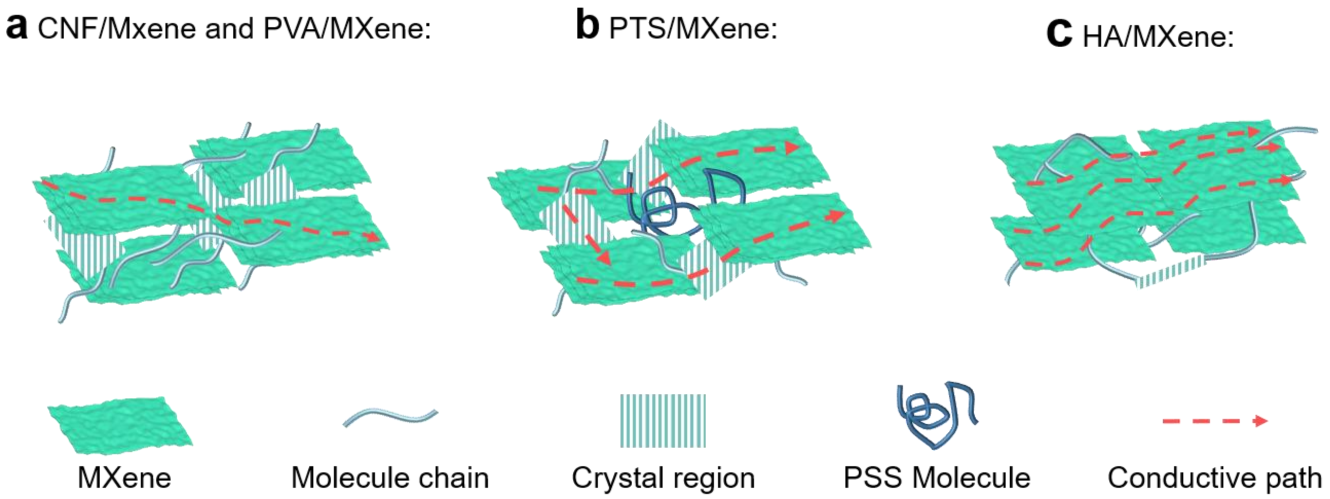

Figure S17. (a) The microstructure illustration of CNF/MXene paper, PVA/MXene paper, (b) PTS/MXene paper, and (c) HA/MXene paper.

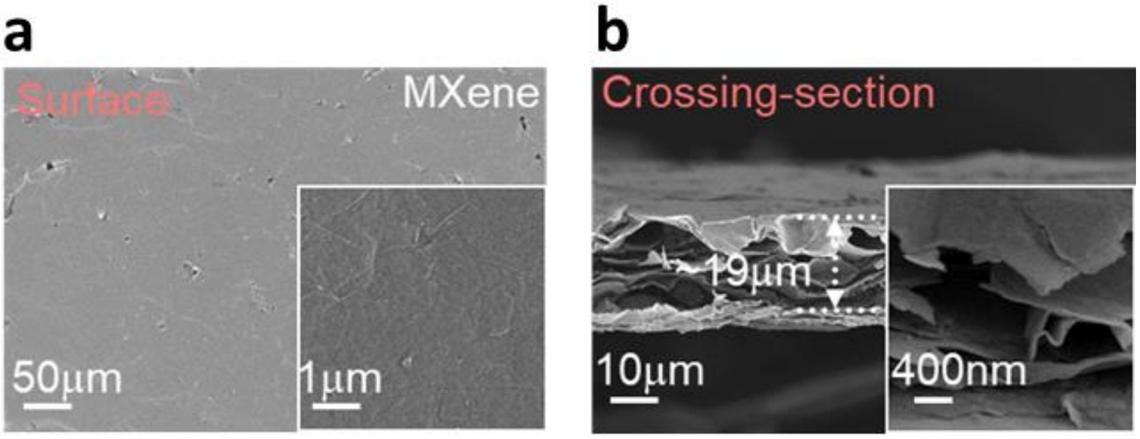

Figure S18. (a, b) SEM images of surface and crossing-section of MXene paper with different magnifications.

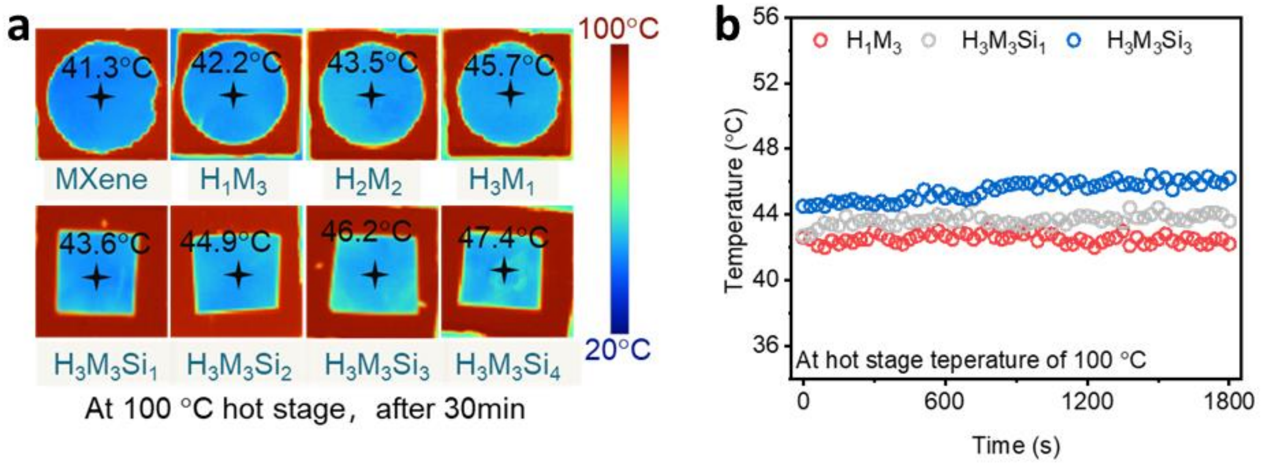

Figure S19. (a) IR thermal images and (b) temperature curves of MXene, HM and HMSi film cover on the 100 °C hot stage.

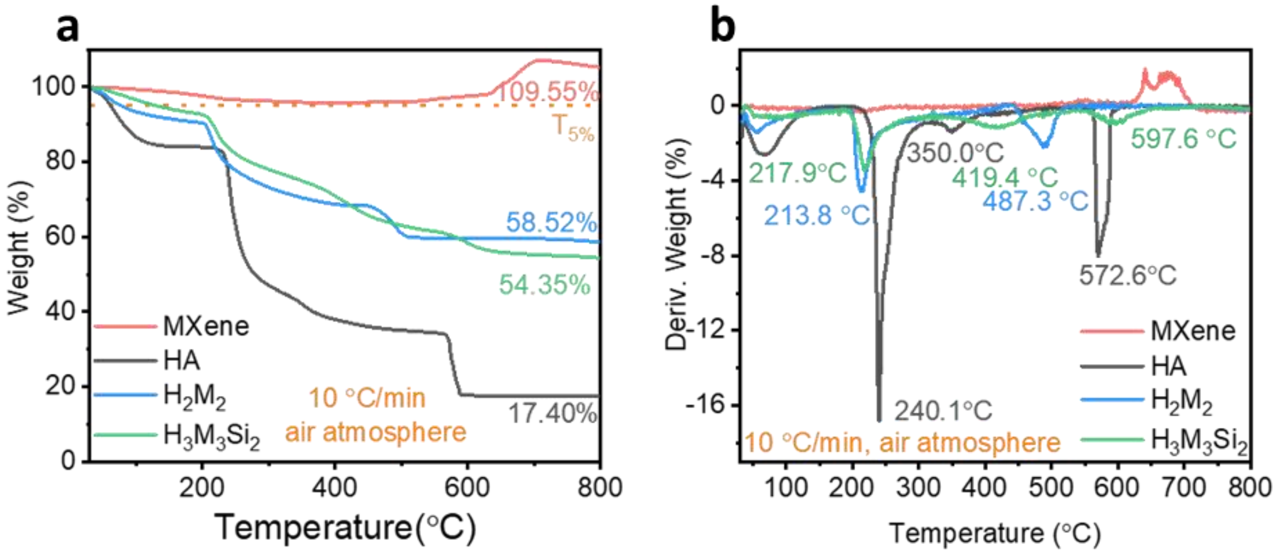

Figure S20. (a) TGA results and (b) corresponding DTG curves of MXene, HA, H_2M_2 and $H_3M_3Si_2$ papers.

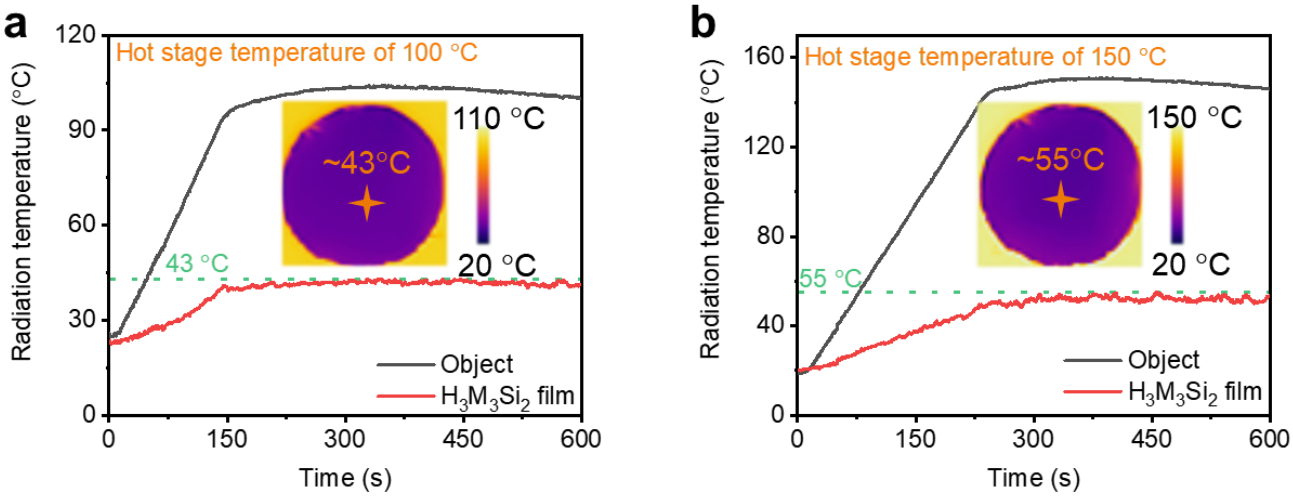

Figure S21. (a-b) Thermal camouflage behavior of $H_3M_3Si_2$ papers under various temperatures and corresponding IR images.

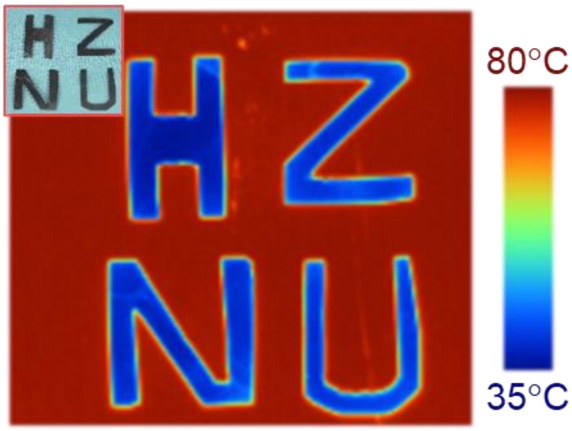

Figure S22. Digital and IR thermal images of "HZNU" letters cut from $H_3M_3Si_2$ film directly.

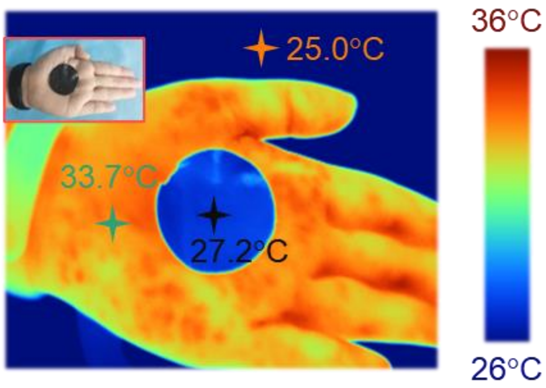

Figure S23. Digital and IR thermal images of palm covered by $H_3M_3Si_2$ film.

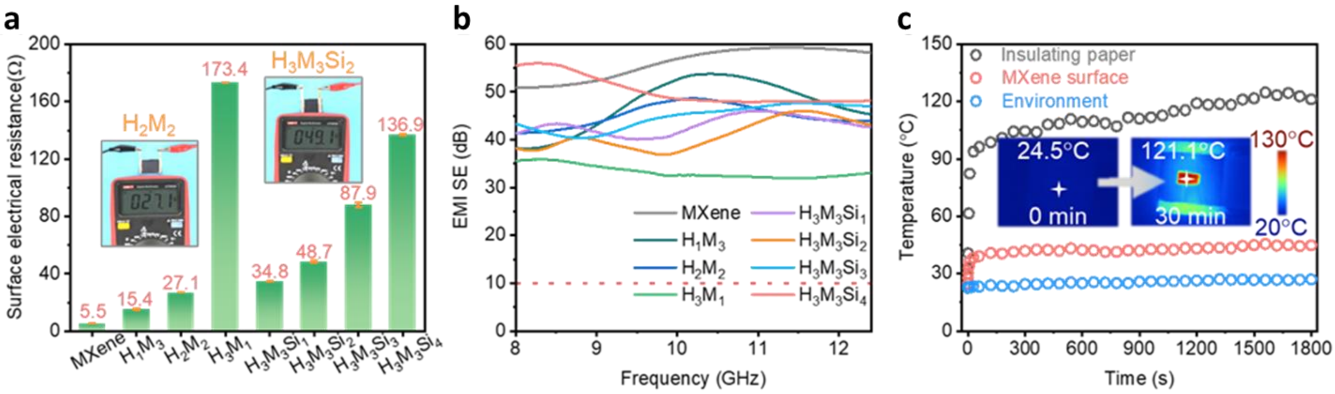

Figure S24. (a) Surface electrical resistance and EMI SE of MXene and (b) various MXene nanocomposites papers. (c) Disguised Joule heating performance of $H_3M_3Si_2$ paper at 3 voltages.

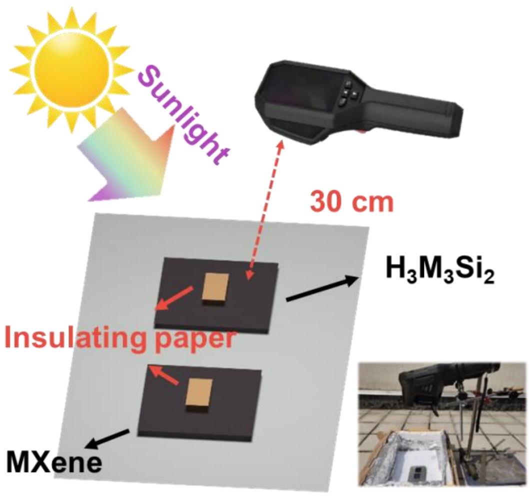

Figure S25. Schematic illustration for thermal camouflage test of $H_3M_3Si_2$ paper in the piratical environment.

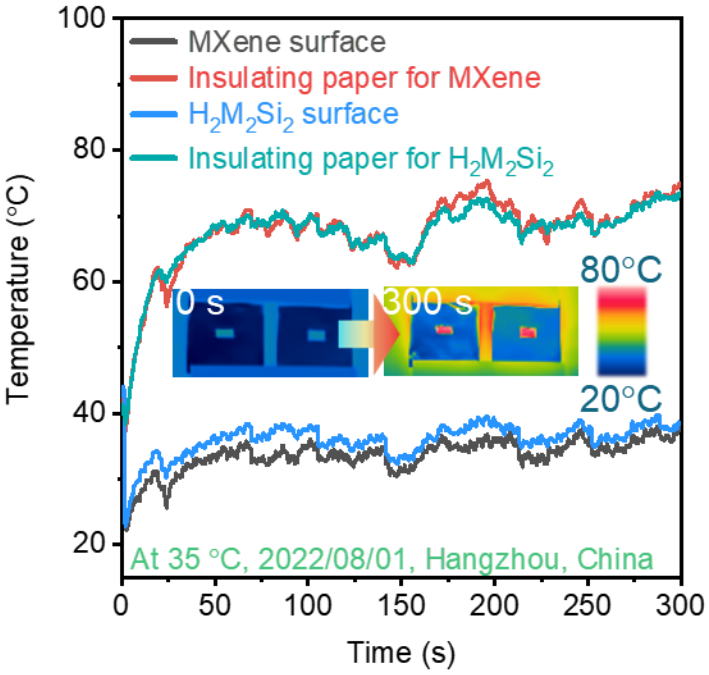

Figure S26. Evaluating thermal camouflage performance of $H_3M_3Si_2$ paper in the sun environment (summer).

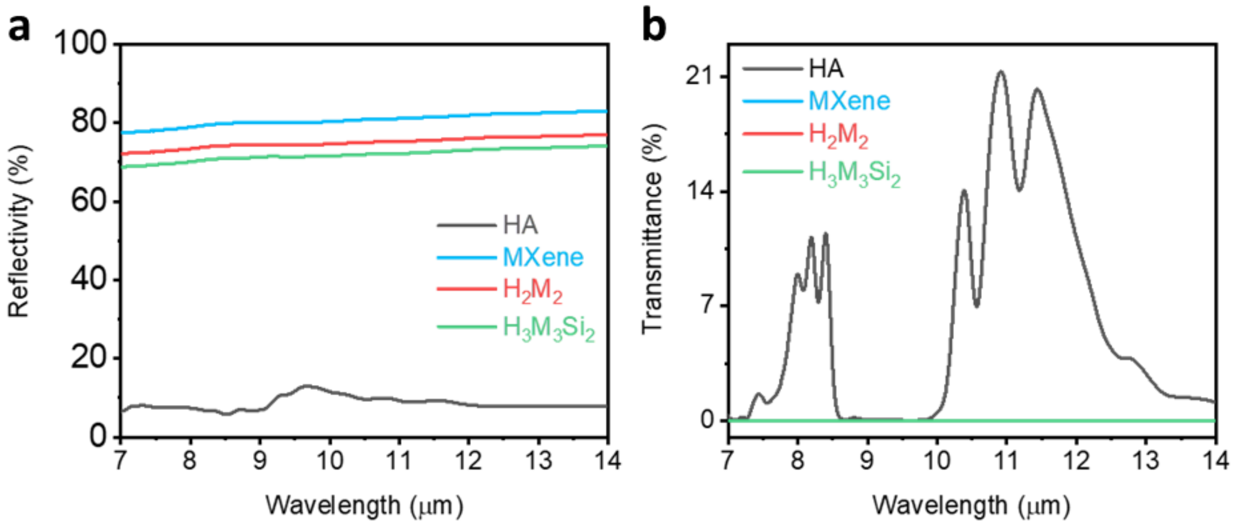

Figure S27. (a) Mid-IR reflectivity and (b) transmittance of HA, MXene, H_2M_2 and $H_3M_3Si_2$ papers.

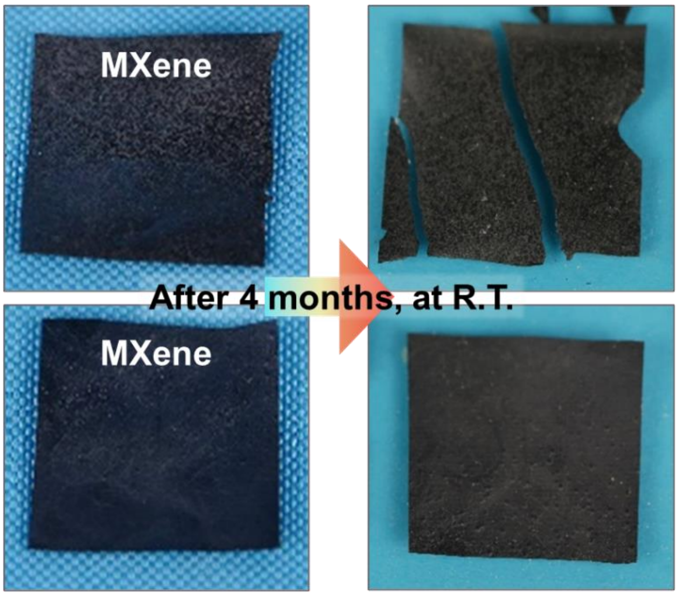

Figure S28. Long-term oxidation measure of MXene-based papers: digital.

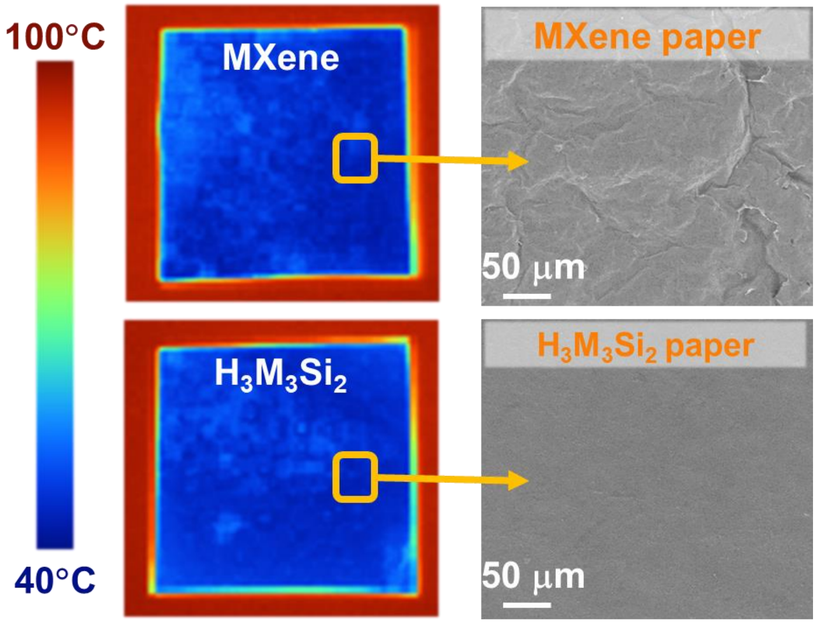

Figure S29. IR thermal images and SEM images of MXene-based papers before long-term oxidation measure.

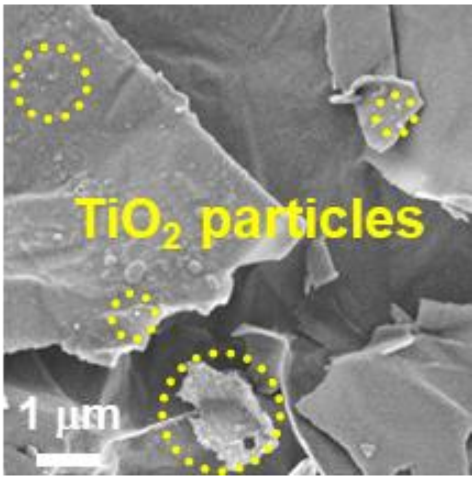

Figure S30. SEM image of the MXene paper surface after 4 months at room-temperature storage.

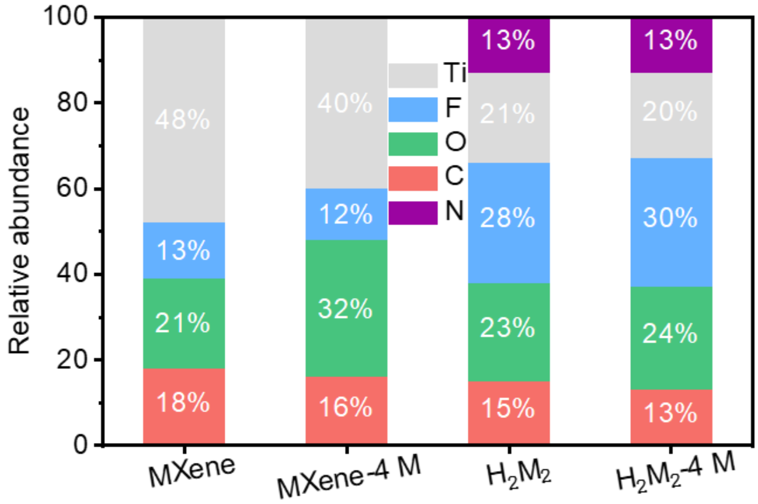

Figure S31. The element ratio of MXene paper surface and $H_3M_3Si_2$ paper surface before and after 4 months at room-temperature storage.

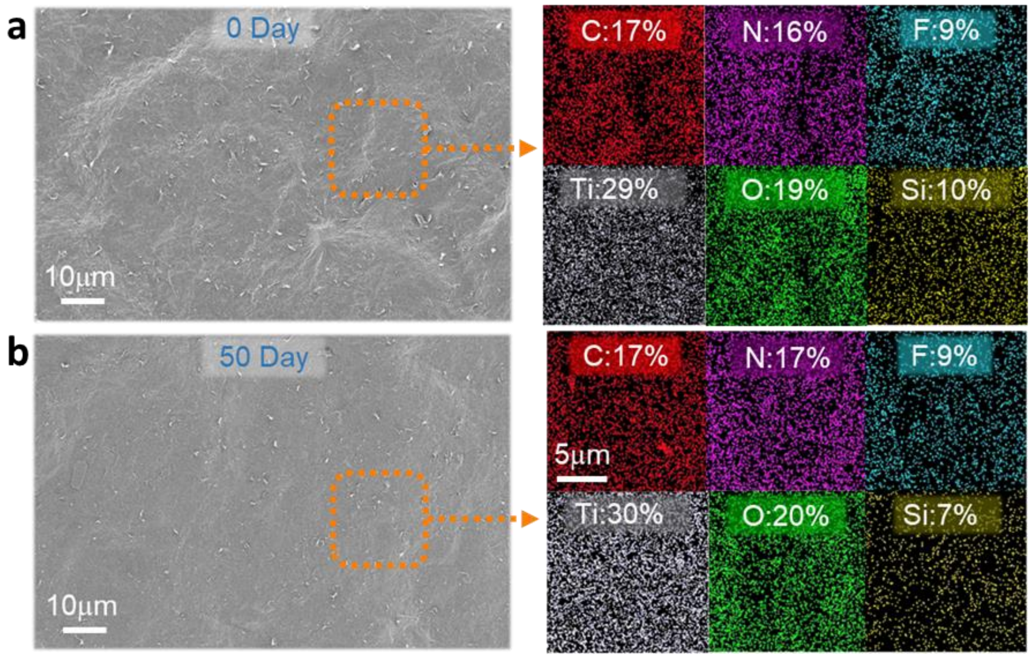

Figure S32. SEM images and Mapping tests of $H_3M_3Si_2$ paper (a) before and (b) after immersed in seawater for 50 days.

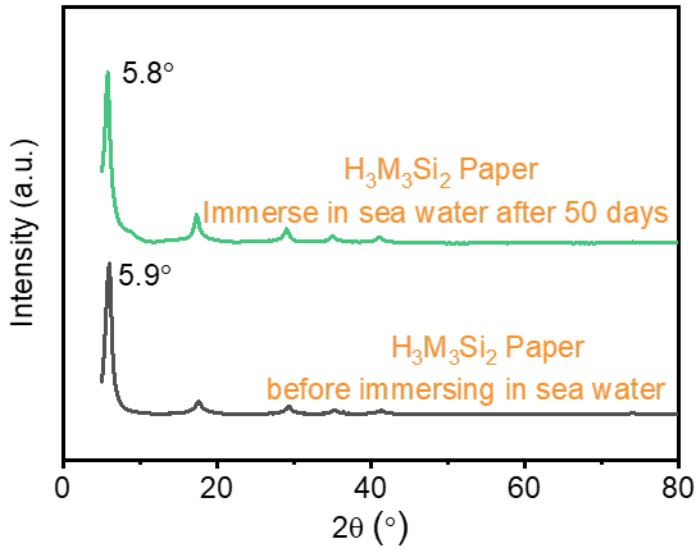

Figure S33. XRD patterns of $H_3M_3Si_2$ paper (a) before and (b) after immersed in seawater for 50 days.

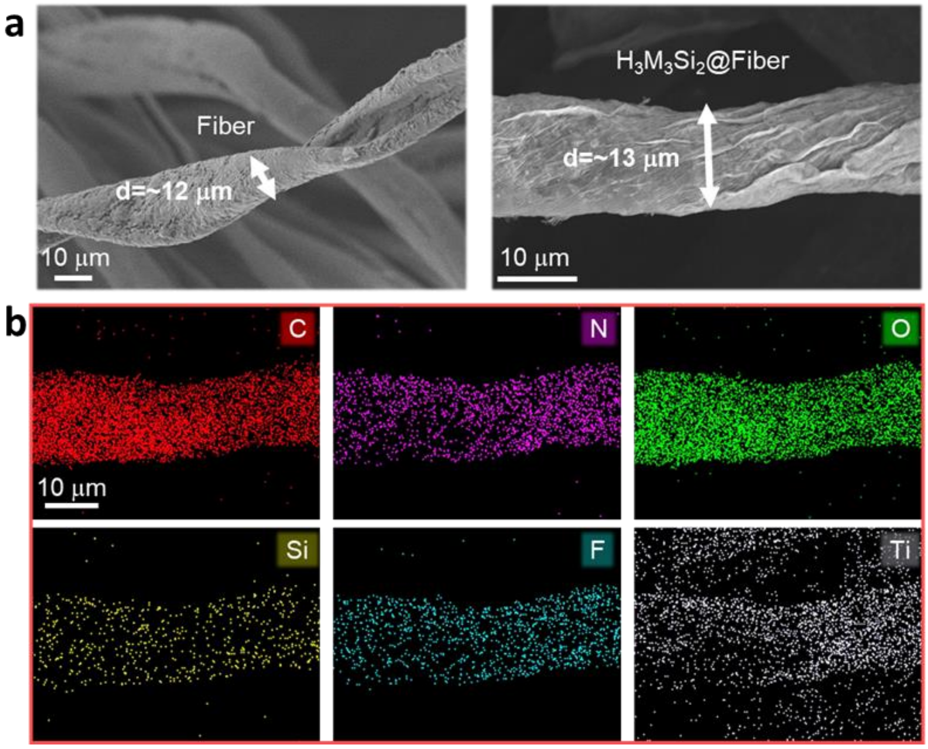

Figure S34. (a) SEM images of fiber (left) and $H_3M_3Si_2$@fiber (right). (b) Mapping test (EDS) of $H_3M_3Si_2$@fiber.

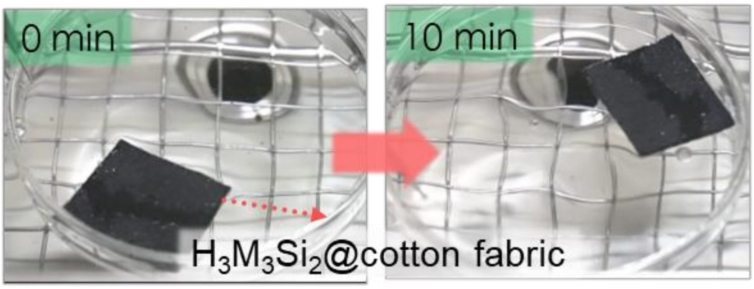

Figure S35. Treating $H_3M_3Si_2$@cotton fabric under ultrasonic circumstances.

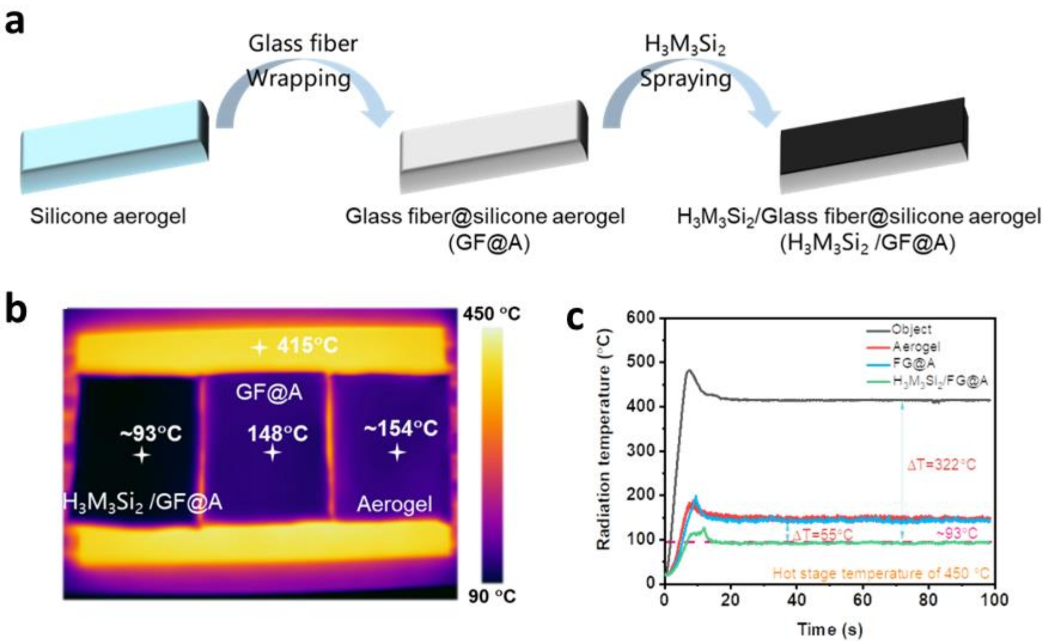

Figure S36. Combining low emissive material with thermal insulation aerogel. (a) The preparation of $H_3M_3Si_2$/Glass fiber@silicone aerogel. (b) Comparison of thermal camouflage property of various samples on the 450 °C hot stage， and (c) corresponding to radiation temperature-time curves.

Table S1. A comparison of comprehensive properties with different MXene-based materials.

**Film type** [a]	**Processing condition** [b]	**IR emissivity**	**Oxygen tolerant** [c]	**Salt water resistant**	**Large preparation**	**Ref.**
MXene	Vacuum-assisted filtration	~0.19	NM	NM	NM	[1]
MXene	Blade coating	~0.14	NM	NM	NM	[2]
MBM(Ni)	LbL assembly, Vacuum-assisted filtration	~0.1	NM	NM	NM	[3]
MP_1P_2	LbL assembly, spraying	~0.5	NM	NM	NM	[4]
SDM	Soak assembly，doctor blade cast	~0.15	√	NM	√	[5]
PET-ITO@MXene	LbL assembly, spraying	~0.247	NM	NM	NM	[6]
MGM-7	LbL assembly, vacuum-assisted filtration	~0.12	NM	NM	NM	[7]
p-MXene-20	Mechanical mixing, vacuum-assisted filtration	~0.278	√	NM	√	[8]
MXene/nanoPE	LbL assembly, spraying	~0.176	NM	√	√	[9]
MXene-TOCNF (50%MXene)	Mechanical mixing, blade coating	~0.562	NM	NM	√	[10]
**$H_3M_3Si_2$** （37.5%MXene）	Mechanical mixing, vacuum-assisted filtration	~0.29	√	√	√	***This work***

[a] MBM: MXene/BP/Ni-MXene; MP_1P_2: polyvinyl alcohol@phase change capsules/polylactic acid/MXene; SDM: MXene/$CaCl_2$/$Na_2B_4O_7$; PET: polyethylene terephthalate; ITO: indium tin oxide; MGM: MXene/graphene oxide/MXene; *p*-MXene: polydopamine-MXene; PE: polyethylene; ML: multi-layer.

[b] LbL: layer by layer.

[c] NM: not mentioned.

References

[1] L. Li, M. K. Shi, X. Y. Liu, X. X. Jin, Y. X. Cao, Y. Y. Yang, W. J. Wang, J. F. Wang, *Adv. Funct. Mater.* **2021**, 31, 2101381.
[2] H. Ma, J. Wang, J. Wang, K. Shang, Y. Yang, Z. Fan, *Diamond Relat. Mater.* **2023**, 131, 109587.
[3] C. Wen, B. Zhao, Y. Liu, C. Xu, Y. Wu, Y. Cheng, J. Liu, Y. Liu, Y. Yang, H. Pan, J. Zhang, L. Wu, R. Che, *Adv. Funct. Mater.* **2023**, 33, 2214223.
[4] X. Li, X. Sheng, Y. Fang, X. Hu, S. Gong, M. Sheng, X. Lu, J. Qu, *Adv. Funct. Mater.* **2023**, 33, 2212776.
[5] S. Wan, X. Li, Y. Chen, N. Liu, S. Wang, Y. Du, Z. Xu, X. Deng, S. Dou, L. Jiang, Q. Cheng, *Nat. Commun.* **2022**, 13, 7340.
[6] X. Y. Du, X. X. Li, Y. X. Zhang, X. Y. Guo, Z. J. Li, Y. X. Cao, Y. Y. Yang, W. J. Wang, J. F. Wang, *Nano Research* **2022**, 16, 3326.
[7] Y. Zhang, L. Li, Y. Cao, Y. Yang, W. Wang, J. Wang, *Mater. Horiz.* **2023**, 10, 235.
[8] Z. Deng, L. Li, P. Tang, C. Jiao, Z. Z. Yu, C. M. Koo, H. B. Zhang, *ACS Nano* **2022**, 16, 16976.
[9] M. Shi, M. Shen, X. Guo, X. Jin, Y. Cao, Y. Yang, W. Wang, J. Wang, *ACS Nano* **2021**, 15, 11396.
[10] S. Feng, Y. Yi, B. Chen, P. Deng, Z. Zhou, C. Lu, *ACS Appl. Mater. Interfaces* **2022**, 14, 36060.
